# Novel interactions of the von Hippel-Lindau (pVHL) tumor suppressor with the CDKN1 family of cell cycle inhibitors

**DOI:** 10.1038/srep46562

**Published:** 2017-04-20

**Authors:** Giovanni Minervini, Raffaele Lopreiato, Raissa Bortolotto, Antonella Falconieri, Geppo Sartori, Silvio C. E. Tosatto

**Affiliations:** 1 Department of Biomedical Sciences, University of Padova, Viale G. Colombo 3, 35121, Padova, Italy; 2 CNR Institute of Neuroscience, Padova, Viale G. Colombo 3, 35121, Padova, Italy

## Abstract

Germline inactivation of the von Hippel-Lindau (VHL) tumor suppressor predisposes patients to develop different highly vascularized cancers. pVHL targets the hypoxia-inducible transcription factor (HIF-1α) for degradation, modulating the activation of various genes involved in hypoxia response. Hypoxia plays a relevant role in regulating cell cycle progression, inducing growth arrest in cells exposed to prolonged oxygen deprivation. However, the exact molecular details driving this transition are far from understood. Here, we present novel interactions between pVHL and the cyclin-dependent kinase inhibitor family CDKN1 (p21, p27 and p57). Bioinformatics analysis, yeast two-hybrid screening and co-immunoprecipitation assays were used to predict, dissect and validate the interactions. We found that the CDKN1 proteins share a conserved region mimicking the HIF-1α motif responsible for pVHL binding. Intriguingly, a p27 site-specific mutation associated to cancer is shown to modulate this novel interaction. Our findings suggest a new connection between the pathways regulating hypoxia and cell cycle progression.

Hypoxia is a common feature shared by the most active tumors, characterized by unregulated development and malignant progression^1^. The complex reactions forming the hypoxia response are mediated by the hypoxia-inducible factor HIF-1α, a transcription factor regulating numerous genes encoding proteins involved in the oxidative metabolism, energy production, cell cycle regulation, as well as red blood cell and vascular endothelial growth factor (VEGF) homeostasis^2^,^3^,^4^. At normal oxygen concentrations, the PHD (prolyl-4-hydroxylase domain) enzymes^5^,^6^ catalyze hydroxylation of two specific HIF-1α prolines P402 and P564 in the N- and C-terminal oxygen-dependent domains (NODD and CODD). Hydroxylated HIF-1α is rapidly targeted for proteosomal degradation by the von Hippel-Lindau tumor suppressor protein (pVHL), an E3 ubiquitin ligase complex substrate recognition element^7^. Hypoxia inhibits PHD activity, impairing pVHL recognition and thereby promoting HIF-1α stabilization^8^. Once stabilized, HIF-1α is translocated to the nucleus, where it activates hypoxia response elements (HRE) promoting hypoxia-regulated gene expression^9^. Deregulation of this network is known to predispose to cancer onset, e.g. in von Hippel-Lindau syndrome, an inherited cancer syndrome characterized by the formation of tumors and cysts in different tissues^10^. Hypoxia is also important in regulating senescence^11^. Programmed cellular senescence is a physiological response evolved to limit the proliferation rate of normal mammalian cells^12^. A cell reaching the so-called Hayflick limit ceases or strongly reduces proliferation, while its metabolism is preserved. Under physiologic conditions, cellular senescence is regulated by many stimuli^13^, including oncogene activity, telomere shortening, oxidative stress and DNA damage. Hypoxia-dependent cellular senescence is also thought to have a critical role in normal tumor suppressor response^11^,^14^, modulating early malignant transformation^15^ and drug-resistance^14^. In particular, hypoxic induction of cell cycle arrest is linked to HIF-1α dependent transcription of the cyclin-dependent kinase inhibitors p21 (CDKN1A)^16^ and p27 (CDKN1B)^17^. Together with p57 (CDKN1C), these form a small family of kinase inhibitors playing important roles in negative regulation of the cell cycle^18^. It is well known that p21 mediates G1 growth arrest^19^ and its transcription is mainly regulated by the tumor suppressor p53 in response to DNA damage^20^. HIF-1α dependent transcription seems to regulate the activation of a specific genetic program designed to slow down the cell cycle in a p53-independent fashion, with marked progression into S phase during hypoxia rather than apoptosis^17^. In parallel, the role of p53 in the regulation of HIF-1α is intriguing. Under prolonged hypoxia, p53 accumulates in the cell yielding repression of HIF-1α transcriptional activity^21^. However it is also thought that a ternary complex between p53, HIF-1α and Mdm2 may promote pVHL-independent degradation of HIF-1α and modulation of p53 activity^22^. Functional connections between hypoxia response and cell cycle regulation are reinforced by recent evidence linking pVHL and p14ARF^23^,^24^, a modulator of Mdm2 function^25^ arising from an alternative reading frame product of the CDKN2A locus encoding the p16ink4a1 tumor suppressor^26^. Here, we describe a novel interaction between pVHL and the CDKN1 inhibitor family. *In silico* sequence, structure and interaction analyses have been complemented with yeast two-hybrid and mammalian cell assays to define the molecular details driving this novel interaction. A conserved region shared among CDKN1 members was found to be responsible for the interaction, with at least one cancer-related mutations able to affect binding.

## Results

### *In silico* investigation of the hypoxia response and apoptotic pathways suggests that pVHL and the CDKN1 protein family could be physically linked

**Figure 1:**
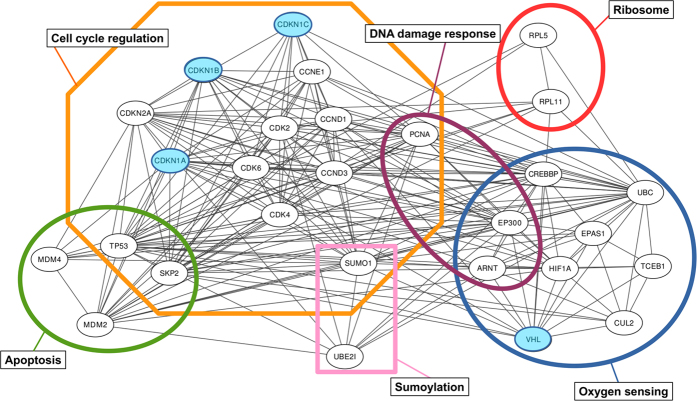
Prediction of pVHL/CDKN1 interactions. Protein-protein interaction network generated with STRING^26^. Functional connections between the pVHL and proteins involved in cell cycle regulation. Connections between nodes represent experimental evidence for interaction. Colored boxes group proteins participating in the same pathway or sharing similar function, e.g. ribosomal proteins. pVHL and CDKN1 are marked in blue.

At the pathway level, pVHL and p53 are thought to associate^27^. To shed light on the functional connection between hypoxia response and cell cycle regulation, an interaction network centered around the proteins pVHL, p53 and CDKN1s was generated with STRING^28^ (Fig. 1). Two almost functionally overlapping pVHL isoforms are reported^29^,^30^,^31^ (pVHL30 and pVHL19, respectively). Here, both isoforms are collectively referred to as pVHL where not explicitly mentioned in the text. The resulting network is composed of 28 nodes connected by 155 edges, with an average node degree of 11.1 and a clustering coefficient of 0.671. The expected number of edges for a similarly populated network composed of random nodes is estimated to be 87. This finding suggests the proteins forming the network should be at least partially biologically connected as group. The network can be divided into six different clusters representing different biological processes (Fig. 1). Proteins directly involved in cell cycle regulation (e.g. the CDKN1 family) form the largest cluster, with 12 nodes. Three smaller clusters (4, 3 and 2 nodes) account for proteins involved in apoptosis, DNA damage response and sumoylation and share nodes with the largest cluster. The connection among these clusters is expected considering the functional role of the CDKN1 family in regulating cell cycle progression^18^. Less obvious is their connection with the second largest cluster (9 nodes), which accounts for proteins involved in oxygen sensing and DNA damage response. Functional enrichment in GO^32^ terms shows “regulation of transcription from RNA polymerase II promoter in response to hypoxia” (GO:0061418) as the biological process best describing the entire network (count in gene set 10, false discovery rate 4.66 e^−18^). Interactions between pVHL and RNA polymerase II subunits are already known^33^,^34^. In general, the functional connection with RNA polymerase II subunits seems to have multiple functional roles. pVHL is thought to modulate Rpb1^33^ expression and is necessary for the oxidative-stress-induced interaction of Rpb1 with DNA^33^, as well as to suppress hsRPB7-induced VEGF promoter transactivation^34^. Considering network topology and connectivity, the data supports a strong functional link between oxygen sensing and cell cycle regulation. Further, pVHL was also proposed to mediate a HIF-1α-independent senescence program^35^, with p27 being upregulated in pVHL null cells. Since large interactome studies of binary protein-protein interactions reveal novel functional interactions among interactors-of-interactors^36^, we decided to address the possible connection between pVHL and the CDKN1 family experimentally.

### pVHL is able to interact with all CDKN1 proteins in Y2H and co-IP experiments

**Figure 2:**
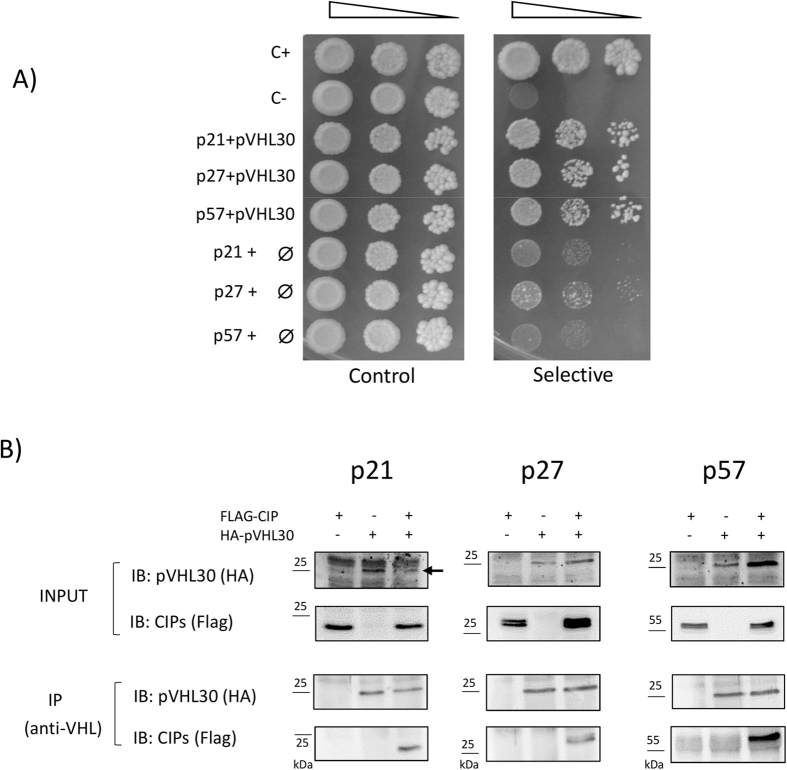
Validation of pVHL/CDKN1 interactions. (**A**) Yeast two hybrid (Y2H) assay of pVHL binding to the CDKN1 proteins. Serial dilutions of yeast cells were spotted on both permissive (*left*) and selective (*right*) media, and incubated for several days at 30 °C. The assayed interaction is shown on the left, with ∅ used for an empty vector (i.e. negative control). C+ and C− are entirely positive and negative controls. The image is representative of four independent experiments, each with 2–3 different clones analyzed. (**B**) Human HEK293T cells were transiently transfected with plasmids overexpressing Flag-tagged CDKN1 proteins and/or HA-tagged pVHL30 protein, as indicated on the top row. Recombinant proteins have been revealed in total cell lysates (*Input*) by immunoblotting with either anti-HA, or anti-Flag antibodies. Upon pVHL immunoprecipitation with a specific antibody, presence of the CDKN1 proteins in the immunoprecipitates (*IP*) was finally verified using the anti-Flag antibody (*bottom panel*). In the p21 panel (*Input*), an arrow indicates the band corresponding to pVHL30, which is partially confused by surrounding unspecific signals.

The protein-protein interaction between pVHL and members of the CDKN1 family (p21, p27 and p57) has been investigated by a genetic two-hybrid system in yeast cells (Y2H). As shown in Fig. 2A, yeast cells expressing either p21, p27, or p57 alone in selective medium almost failed to grow, as well as with pVHL30 only (Supplementary Figures S1–S3). Cell growth was markedly improved in presence of pVHL30, indicating that the proteins were able to associate *in vivo*. Our data further suggested a common pVHL interaction site with CDKN1 protein sequences. Co-immunoprecipitation experiments in human cells were performed to confirm the interactions in a more physiological context. A series of plasmids able to over-express either HA-tagged pVHL30 protein, or the Flag-tagged CDKN1 proteins in mammalian cells were constructed. Recombinant plasmids were used to transiently transfect HEK293T cells and perform Co-IP assays from total cell lysates using a specific anti-VHL antibody^37^,^38^. Our data (Fig. 2B) indicates that all three CDKN1 proteins were able to interact with pVHL30, as demonstrated by their presence in the immunoprecipitate revealed with the anti-Flag antibody (bottom panels). Taken together, the experiments show that these proteins can form at least binary complexes in human, and notably kidney cells.

### A conserved motif in the CDKN1 domain resembles HIF-1α, and MD simulations suggest the pVHL β-domain to drive the interaction

**Figure 3:**
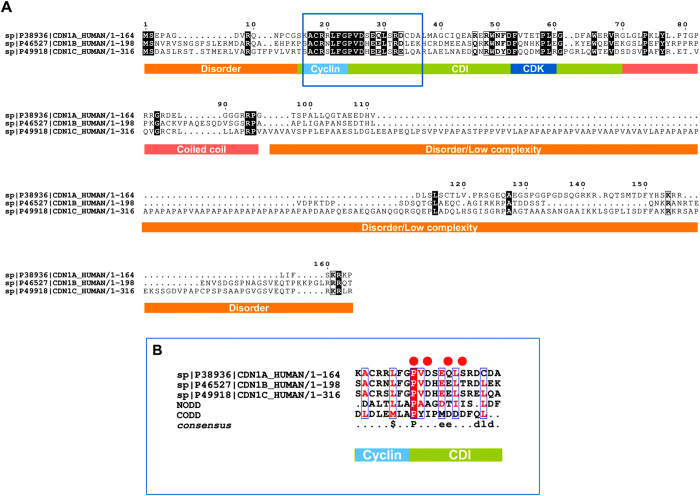
Overview of the CDKN1 sequence features. (**A**) Multiple sequence alignment and feature analysis of the CDKN1 protein family. The functional motif organization is presented as a colored bar below. (**B**) Close-up of sequence conservation between the CDI domain and NODD/CODD motifs of HIF-1α. The CODD-like region localizes to the CDI domain (green bar), partially overlapping the cyclin A recognition element (light blue). Red dots represent cancer-related mutations in the CODD-like region of p27. The consensus sequence is shown below, with $ used for hydrophobic residues.

The CDKN1 proteins share a conserved 48-residue domain located at the N-terminus identified in Pfam as the CDI protein family (pfam02234). Multiple sequence alignment showed relevant (64%) sequence similarity with the CODD^7^ motif of HIF-1α, a value high enough to assume conserved features among the sequences. In particular, the CDI seems to be chemically compatible with amino acids 556–574 of HIF-1α (Fig. 3), a functional region responsible for the interaction with pVHL^7^. Data in the literature^39^,^40^,^41^ as well as secondary structure prediction of CDI performed with FELLS^42^ suggested this short segment to adopt random coil structure when not in complex to cyclin A. Combining these findings, we asked whether CDI may sustain the interaction with pVHL in a HIF-1α like fashion. Inspection of the p27 crystal structure (PDB identifier: 1JSU) showed the region partially overlapping the putative CODD motif, forming a short coil (residues 25–37) binding cyclin A. This finding reinforced the idea that this segment may adopt an extended conformation able to interact with pVHL under physiological conditions. Intriguingly, four cancer-related p27 mutations were found to affect the CODD-like region^43^. Whereas multiple experimental evidences describe the pVHL/CODD interaction^7^,^44^, no structural data is available for the interactions investigated here. The corresponding p27 region (p27-CODD-like, residues 27–51) was modeled, using the HIF-1α CODD crystal structure (PDB code: 1LM8, chain H) as template. The obtained model was used to perform molecular dynamics (MD) simulations to investigate whether such a motif is able to sustain the interaction. Our simulations showed p27-CODD-like bound in a stable way to pVHL after 50 ns. The p27-NT backbone was predicted to assume a β-sheet-like conformation binding the fourth pVHL β-strand through 2 hydrogen bonds with pVHL residues I109 and H110. The interaction was further sustained by a salt bridge between p27-E40 and pVHL-R107 as well as an additional van-der-Waals interaction between the pVHL-H115 backbone and p27-G34. Additional investigation with RING 2.0^45^ showed that p27-E40 is able to form interactions with other residues on the same interface of the pVHL β-domain (Supplementary Figure S4). The MD simulations also predicted the interaction to be stable in the absence of Elongin B and Elongin C, suggesting a possible proteasome-independent function. Although MD simulations are not necessarily representative of physiological conditions, the *in silico* data collectively suggest binding between pVHL and the CODD-like domain of the CDKN1 proteins.

### Dissection of the pVHL-CDKN1 interacting regions

**Figure 4:**
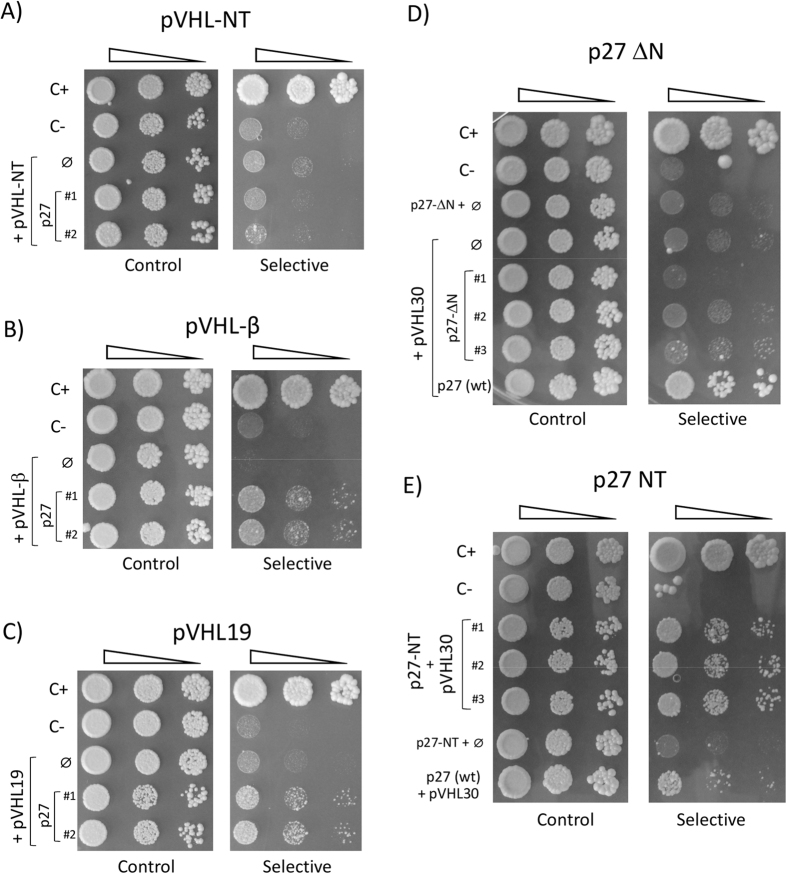
Y2H dissection map of pVHL binding to p27. Yeast two hybrid (Y2H) assays are shown of pVHL binding to p27, including fragments of either protein. Serial dilutions of yeast cells were spotted on both permissive (*left*) and selective (*right*) media, and incubated for several days at 30 °C. The assayed interaction is shown on the left, with ∅ used for an empty vector (i.e. negative control). C+ and C− are entirely positive and negative controls. The image is representative of four independent experiments, each with 2–3 different clones analyzed. (**A**) Y2H assay of pVHL-NT (residues 1–53) with p27 shows no growth on selective medium. (**B**) Y2H assay of pVHL-β (residues 54–157) with p27 shows growth on selective medium. (**C**) Y2H assay of pVHL19 (residues 54–213) with p27 is indicative of their binding. (**D**) Removal of the first 60 residues of p27 (p27-ΔN) abolishes interaction with pVHL30. Plates were incubated at 30 °C for longer time (8 days), to confirm the absence of yeast growth. (**E**) pVHL30 were tested for binding by Y2H assay with the p27 N-terminus (NT, residues: 1–60). On selective medium, p27-NT yeast cells display increased growth rate with respect to the full-length protein (wt), possibly reflecting a negative impact of the p27 C-terminus on pVHL binding. Plates were incubated at 30 °C for 4 days.

The regions involved in binding were mapped using the Y2H system to verify the computational results. The pVHL30 protein was first dissected in three parts, N-terminal disordered tail (residues 1–53), β-domain (54–157) and α-domain (158–204). Mutant plasmids expressing the different pVHL fragments were generated and yeast cells transformed to determine binding with each CDKN1 protein. Our results show the pVHL N-terminus not interacting, as yeast cells expressing it together with any CDKN1 protein are unable to grow in selective medium (Fig. 4A, Supplementary Figures S5 and S6). The pVHL β-domain is able to bind any member of the CDKN1 family, as indicated by yeast cell growth (Fig. 4B, Supplementary Figures S5 and S7). The results also confirmed that the pVHL19 isoform, lacking the N-terminus is able to interact with all CDKN1 proteins like the pVHL30 protein (Fig. 4C, Supplementary Figures S5 and S8). The data also suggests that the pVHL α-domain, while not strictly required, may be important for proper CDKN1 binding, possibly by stabilizing the pVHL structure.

The CDKN1 region involved in pVHL binding was mapped based on the conservation shared by the three CDKN1 sequences. Yeast plasmids expressing either their N-terminal tail containing the CDI domain (p27-NT residues: 1–60; p21-NT: 1–49; p57-NT: 1–61) or the corresponding C-terminal moiety lacking the N-terminus (p27-ΔN residues: 61–198; p21-ΔN: 50–164; p57-ΔN: 62–316) were generated to test the effects of CDKN1 binding to pVHL30. As shown in Fig. 4D, loss of the p27 N-terminus clearly disrupts its ability to associate with pVHL30, as yeast cells expressing the C-terminus of p27 were all unable to grow in selective medium. Similar data have been also obtained for both p21 and p57 (Supplementary Figures S9 and S10), strongly supporting the notion that the CDI domain is responsible for CDKN1 binding to pVHL30.

The final demonstration came by directly assaying the interaction between pVHL30 and the N-terminus of each CDKN1 protein. As shown in Fig. 4E (and Supplementary Figure S11), the N-terminus of p27 was able to sustain the growth of pVHL30-expressing yeast cells even more efficiently than full-length p27. Similar results have been also observed for the N-terminus of p57 (Supplementary Figures S5 and S11). Although data on the p21-NT fragment cannot be considered due to unspecific activation of the reporter gene (i.e. auto-activation, Supplementary Figure S9) our results suggest that removal of the CDKN1 C-terminus may have positive effects on pVHL binding, as judged by increased yeast cell growth in selective medium expressing the N-terminus.

### Pathological p27 mutations may influence pVHL binding

**Figure 5:**
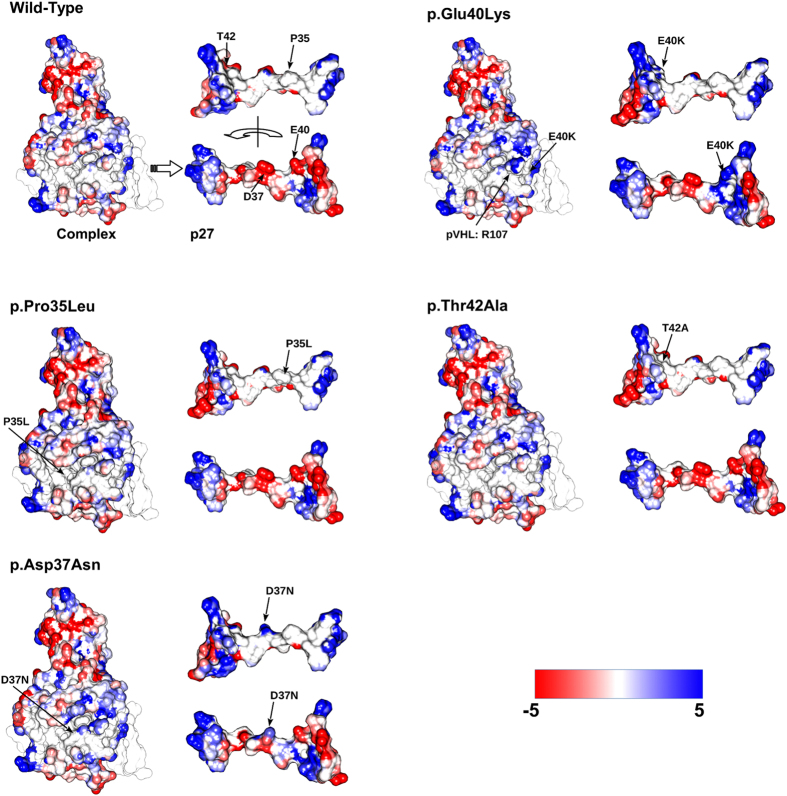
*In silico* prediction of mutations putatively affecting pVHL/p27 binding. Solvent accessible surface representation of a predicted pVHL/p27-NT complex colored by electrostatic potential. Blue represents positively charged areas, red negative. For each mutation, the full complex is presented with p27 alone next to it. Both the front (accessible surface) and rear (interaction interface) views are shown. The electrostatic potential was generated with Bluues^47^.

The predicted binding site between pVHL and the CODD-like p27 motif, shared by all CDKN1 proteins, has been further tested by inserting several N-terminal p27 missense mutations. The substitutions P35L, D37N, E40K and T42A (Fig. 3B) have been selected considering the conservation of the CODD motif, their position on the interaction surface as well as their pathological relevance in cancer^43^, i.e. hematological malignancies compatible with the deregulation of the pVHL/HIF-1α axis. None of these had been characterized with respect to the normal function of p27 as CDK inhibitor. *In silico* replacements of both D37N and T42A were predicted by NeEMO^46^ to stabilize (or have a negligible impact) on the pVHL/p27 binding moiety (Table 1), while the contrary is predicted for E40K and partially for T42A. MD simulations provided similar predictions. Notably, trajectory inspection suggests P35L to bind slightly better as it is facing a pVHL hydrophobic pocket. Conversely, E40K is predicted to negatively perturb the interaction, which could generally reflect the impact of charge inversions at the pVHL/p27 binding interface. To better address this behavior, we characterized the electrostatic properties of the putative pVHL/p27 complex wild-type in respect to the complexes formed with mutants p27 using Bluues^47^ (Fig. 5). As expected, almost no variation in the solvation energy is predicted for P35L (−0.89 kJ/mol), with a modest variation predicted for D37N (−8.83 kJ/mol). Aspartic acid 27 is exposed to solvent and a change to asparagine is predicted to stabilize the complex little. Conversely, E40K is predicted to markedly destabilize the interaction. During MD simulations, we observed the formation of a salt bridge between p27 E40 and pVHL R107. A change in lysine clearly abolishes this interaction, suggesting a repulsive effect. We predict a difference in electrostatic solvation energy of 183.33 kJ/mol for this mutant, a value high enough to destabilize the complex. A slightly positive variation (3.58 kJ/mol) is predicted instead for T42A. As T42 is located in a partially hydrophobic pocket at the pVHL/p27 interface, we believe it should have a modest impact on complex formation. APBS^48^ was used to quantify the binding free energy electrostatic component, required for pVHL/p27 complex formation (Table 1). The binding free energy predicted for the wild-type complex is Δ_bind_G −22.07 kcal/mol, a favorable value for complex formation. Similar values are predicted for mutants D37N and T42A (Δ_bind_G −22.56 and −20.65 kcal/mol, respectively) and Δ_bind_G −28.05 kcal/mol is predicted for P35L. Instead, a positive value is predicted for E40K (Δ_bind_G 40.86 kcal/mol). Collectively, these findings suggest that E40K is able to disturb the pVHL/p27 interaction, while a modest effect is predicted for the other mutants.

**Figure 6:**
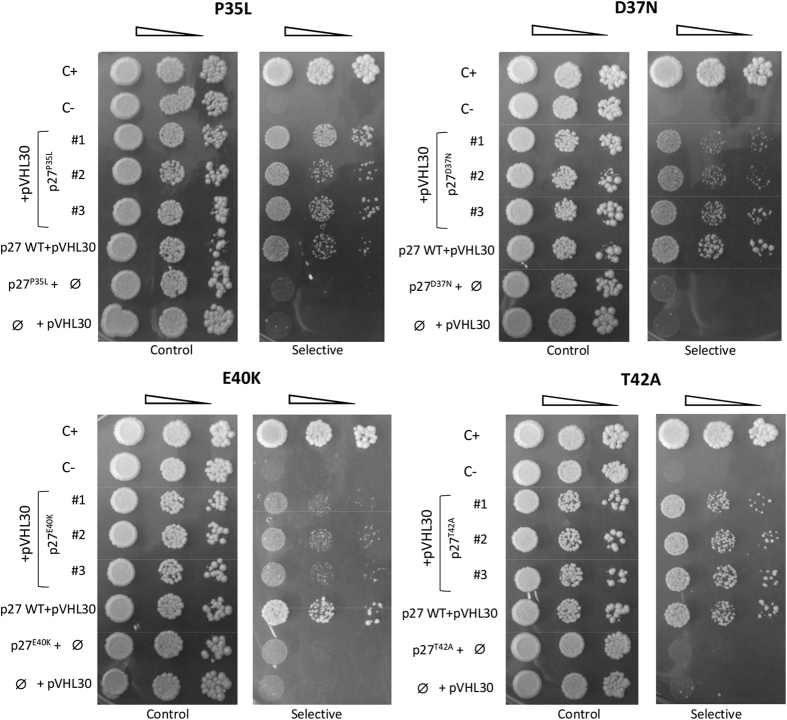
Effects p27 mutagenesis on pVHL binding. *Y2H analysis of the p27 mutations P35L, D37N, E40K and T42A*. Yeast cells co-expressing pVHL30 together with the indicated mutant p27 isoforms were assayed in Y2H. Three independent mutant clones are shown. Yeast cells have been grown in selective medium (right) supplemented with 60 mM 3AT, in order to increase the stringency of the binding assay. In all experiments, C+ and C− are positive and negative controls of the assay, respectively. The images are representative of three independent experiments, where 3–5 clones were analyzed.

The predictions have been then experimentally tested by Y2H assay (Fig. 6). Both p27 T42A and D37N mutants retained the ability to associate pVHL30 almost completely, as indicated by similar yeast cell growth in selective medium. The data further confirm that P35L does not perturb binding, as its introduction did not affect the growth rate of mutant yeast cells. On the contrary, E40K reduced p27 binding to pVHL30, as yeast cells expressing the p27 almost failed to grow in selective medium. The negative effect of the mutation has been confirmed by testing additional clones (Supplementary Figures S12 and S13). Collectively, our results on p27 mutants further support that p27/pVHL binding may be described by the *in silico* model.

## Discussion

The starting point for our work lies in the observation that the Pfam CDI domain (pfam02234), which identifies the cyclin-dependent kinase inhibitor (CDKN1) protein family as sharing common elements with the CODD motif of HIF-1α, involved in its binding with the pVHL protein. We provided evidence that all CDKN1 proteins (p21, p27 and p57) are able to associate with pVHL *in vivo*, as first indicated by Y2H assays and confirmed in mammalian cells by Co-IP. Our data point to a novel connection between the regulation of cell proliferation and transduction of multiple signals related to the HIF-1α and p53-dependent pathways. The functional association with pVHL may have important effects on the CDKN1 proteins, possibly influencing their regulatory functions. Recent findings show regulation of p27, in particular, to be linked with other components of the oxygen sensing pathway, such as PHD3^49^ which is thought to drive cell cycle entry at the G1/S transition by decreasing p27 stability. In this scenario, interaction with pVHL could serve as further modulator of p27 half-life. Our data highlights the common features between the interactions of the pVHL-β domain with both the CDKN1-CDI and HIF1-CODD regions, as supported by dissection of their binding elements. The pVHL/p27 interaction has been modeled *in silico* and investigated with respect to p27 pathological mutations found in COSMIC^43^ and associated with cancer development. Collectively, *in silico* calculations and *in vivo* validations support that the association is maintained under hypoxic conditions, i.e. proline hydroxylation is not necessary, and potentially able to exert their functional roles within the cells. Cell cycle progression, where the CDKN1 proteins act as natural inhibitors may be functionally linked *via* pVHL to the HIF-1α and HIF-2α-dependent pathways, i.e. hypoxia/angiogenesis response, which is particularly relevant in cancer development. It is well known that under prolonged hypoxia, HIF-2α plays a key role in promoting genomic integrity and cell cycle regulation by stimulating c-Myc-mediated activation of cyclin D2 and the E2F1 transcription factor with concomitant repression of p21 and p27^50^. Importantly, since the same pVHL interaction surface may be involved, alternative binding to either HIF-1/2α, or each CDKN1 protein, might be mutually exclusive, and possibly competitive. In this biological context, particularly relevant is the observation that the pVHL/CDKN1s interaction is possible also in the absence of a hydroxylated proline. The pVHL interface B is known to bind different proteins^33^,^34^,^51^ in a CODD-like fashion, however all these interactions require previous hydroxylation of the binding partner. In other words, it can be assumed that these proteins collectively compete with HIF-1α for the same binding interface at physiological oxygen concentrations. In both mild and prolonged hypoxia, PHD-dependent proline hydroxylation is inhibited^5^,^6^, reducing competition for interface B. Very recently, a novel hydroxylation-independent interaction between pVHL and Aurora kinase A (AURKA)^52^, a serine/threonine kinases essential for cell proliferation was reported. In this context, interaction with the CDKN1 protein family may have evolved to allow transmission of a generic hypoxic signal to other signaling pathways, re-using the same pVHL adaptor protein. Intriguingly, the same pVHL region^53^,^54^ mediates its association with over 40 proteins beyond HIF-1/2α^54^, implying multiple factors in dynamic competition to alternatively associate unbound pVHL in the cell.

These associations likely have to be also regulated at the post-translational level and may be related to different signals/pathways, as observed for HIF-1α hydroxylation by the proline hydroxylase (PHD) enzymes. Since yeast cells are devoid of PHD activity, our data strongly suggest that hydroxylation should not be involved in regulating CDKN1 binding, as further supported by the p27 P35L mutant. Experimental evidence suggested a negative impact of the CDKN1 C-terminus on association with pVHL, pointing to regulatory effects on the binding upon introduction of specific post-translational modifications, known to occur within the C-terminal region of the CDKN1 proteins^55^,^56^,^57^,^58^.

Although preliminary correlations between hypoxia response and the CDKN1 proteins have been reported^49^,^59^,^60^, the consequences of their association with pVHL are far from understood. As a member of the VCB complex, pVHL could also regulate CDKN1 degradation, similarly to HIF-1α, although published data on p27 turnover indicate the involvement of the Skp2 system^61^. Interestingly, a direct interaction between Skp2 and pVHL, mediated by its β-region, has been reported to stimulate Skp2 proteasomal degradation, independently from the pVHL-VCB E3-ligase activity^62^. Increased p27 levels have been observed in several pVHL-mutant cell lines^63^, further pointing to their functional association. Future work is needed to address if (and how) binding with pVHL/VCB could impact both stability and function of the CDKN1 proteins in cancer and health. The relative affinity of pVHL binding (i.e. K_d_ values) should be determined. Moreover, mammalian cells should be used to investigate the competitive nature of the binding, to determine which are the functional implications of the protein-protein interactions characterized here. Our results on site-specific mutations, combining both computational and experimental approaches, contribute to shed light on the effects of pathogenic variants towards the association of p27 with pVHL, which could help to clarify the relationship between clinical phenotype and functional defects caused by CDKN1 mutations.

## Materials and Methods

### Interaction network and sequence feature analysis

Amino acid sequences (with UniProt accession numbers in parentheses) for pVHL (P40337), p21 (P38936), p27 (P46527) and p57 (P49918) were retrieved from UniProt^64^ selecting the canonical sequence and visualized with Jalview^65^. Alignment was performed with T-Coffee^66^ using default parameters. Disorder was assessed with MobiDB^67^ and DisProt^41^, while functional domains were retrieved from Pfam^68^ and InterPro^69^. A protein-protein interaction network centered around pVHL and CDKNs was derived from STRING^28^. To maximize data reliability, text-mining and neighborhood interactions were excluded. The first shell of interactors was populated with pVHL, p21, p27 and p57 selecting the corresponding human proteins, and no more than 20 interactors were chosen for the second shell. The default interaction score confidence parameter (0.400) was used and the resulting network analyzed with Cytoscape^70^.

### Molecular dynamics simulations

The 1.8 Å crystal structure of pVHL (PDB identifier: 1LM8)^7^ and the 2.3 Å structure of p27 (PDB identifier: 1JSU)^71^ were used as starting models. The pVHL/p27 complex was constructed by homology modeling using Modeller^72^ through superimposition to the pVHL/HIF-1α complex. All simulations were carried out with GROMACS^73^ using the CHARMM27 force field and the TIP3p explicit solvent model. All simulation runs consisted of 100 conjugate gradient minimization steps, 100 ps in NVT conditions, and 50 ns of classic molecular dynamics simulation at 310 K and 1.01325 bar. Integration was based on the Verlet method^74^ using a 2 fs time step. Trajectories were compared in terms of RMSD and root-mean-square fluctuation (RMSF). RING 2.0^45^ was used to estimate variation in residue interaction network with strict distance thresholds and the “one interaction” option.

### Selection and interpretation of mutations putatively affection pVHL/p27 interaction

Considering both sequence conservation with CODD motif and their specific association with cancer, the following cancer related p27 mutations putatively affecting the pVHL/p27 interaction were retrieved from COSMIC^43^: P35L, D37N, E40K and T42A. The first three localize in or are immediately close to conserved positions, while p.T42A has been chosen because it was found in patients with hematological malignancies^75^. Both driver and passenger mutations affecting the CODD-like region were included in this study due to the novelty of the pVHL/p27 interaction and a lack of specific bibliographic data. Mutations were placed with Bluues^47^ starting from the last MD simulation frame after removal of solvent and ion molecules. Bluues was also used to predict the electrostatic properties of wild-type and mutant p27. Stability was predicted for each mutant with NeEMO^46^. Mutations on the pVHL/p27 interaction were investigated predicting the electrostatic Δ_bind_G values with APBS^48^, a method which evaluates the energy difference between the solvated unbound interactors and the solvated complex.

### Plasmid constructs

The pcDNA3.1-derived plasmids carrying synthetic full-length cDNA sequences coding for the human pVHL30, p21, p27 and p57 proteins were purchased from GenScript (GenEZ plasmids OHu23297, OHu27895, OHu26670 and OHu27234 respectively). Plasmids were used as starting point to transfer the cDNA sequences (full-length or corresponding to the different protein regions) in the pGADT7 and pGBKT7 plasmids (Clontech), used to perform yeast two-hybrid assays. Untagged cDNA sequences were amplified by PCR using specific primers (Supplementary Table S1) carrying 15 nucleotides long 5′ ends corresponding to specific regions surrounding the EcoRI site in the MCS of pGADT7 and pGBKT7 vectors. PCR products were directly cloned in two vectors linearized by digestion with EcoRI using the In-Fusion^®^ HD Cloning Kit (Clontech) following the manufacturer protocols. All recombinant plasmids used in the yeast two-hybrid assays (Supplementary Table S1) are able to express the proteins of interest in fusion with either the DNA binding domain (DBD) or the activation domain (AD) of the Gal4 transcription factor. The cMyc and HA epitopes present in the fusion proteins with the Gal4 DBD and AD, respectively, allow to readily verify the expression of the chimeric proteins in yeast cells. GenScript plasmids have been directly used to overexpress the different CDKN1 proteins as N-terminal FLAG-tagged fusion polypeptides in mammalian cells. The 5′-end of each cDNA was joined in-frame with the sequence coding for the FLAG epitope. The In-Fusion^®^ HD Cloning Kit (Clontech) was used to clone the pVHL30 cDNA in the BamHI/EcoRI sites of pcDNA3.1 vector, with the addition of the HA epitope sequence at the N-terminus. The resulting pVHL30-pcDNA3.1 plasmid was used in mammalian cells to overexpress pVHL30 as N-terminal HA-tagged protein. Yeast plasmids expressing single-residue mutagenized p27 (P35L, D37N, E40K and T42A) were obtained using the QuikChange II XL Site-Directed Mutagenesis Kit (Agilent Technologies), starting from the corresponding wild-type plasmid in a single-round PCR step, following the manufacturer procedures. All sequences cloned in the recombinant plasmids described in this paper have been verified by Sanger sequencing prior to use in the experiments.

### Yeast two-hybrid (Y2H) assays

Interactions between the three CDKN1 family members and pVHL have been investigated by the two-hybrid assay, using the Matchmaker^®^ Gold Two Hybrid System (Clontech) using standard Yeast growth conditions, media and transformation protocols. Experimental conditions to evaluate the positivity of the assay have been first established by following the growth of the Y190 reporter strain in selective medium lacking histidine supplemented with 30 mM 3-AT. In the assays, the same negative and positive internal controls have been always added, represented respectively by the Y190 yeast strain carrying both empty pGBKT7 and pGADT7 plasmids (no binding) and Y190 cells co-transformed with the pGADT7-T (Gal4 AD-SV40 large T-antigen) and pGBKT7–53 (Gal4 DBD-murine p53, fragment 72–390) plasmids (strong binding) provided by the manufacturer. Yeast Y190 cells co-expressing the fusion protein with the Gal4 DBD and AD domains alone, or *vice-versa*, have also been tested to exclude false positivity of the assay due to the auto-activation by Gal4-fusion proteins. Expression in yeast cells of the Gal4-fusion proteins has been checked by Western blot analysis (Supplementary Figure S15), while the functional status of pVHL was verified testing the well-known pVHL/HIF-1α interaction with and without co-expressing PHD3 (Supplementary Figure S16). Multiple (3–5) transformations were generally performed, where (at least) two independent colonies for each experiment were picked, inoculated in liquid medium, and grown to exponential phase. Yeast cells were then serially diluted 10-fold, and spotted on either solid selective medium lacking histidine and containing 30 mM 3-AT, or permissive medium (i.e., supplemented by histidine), to check both cell viability and number. Growth of yeast strains was constantly monitored for 3 to 8 days at 30 °C. When necessary, interactions have been further tested in selective medium supplemented with 60 mM 3-AT, to increase the stringency of the assay by doubling the concentration of the competitive inhibitor. Total yeast proteins have been obtained by TCA-based solubilization of yeast cells, as described in ref. 76, followed by standard Western Blot analysis^77^, by using either anti-HA (Abcam, ab16918), or anti-Myc (Abcam, ab127421) antibodies, to reveal the HA-tagged Gal4AD-pVHL, or the Myc-tagged Gal4BD-CDKN1 proteins, respectively.

### Transfection and co-immunoprecipitation from HEK293T cells

Human kidney HEK293T cells were used for co-immunoprecipitation experiments. Cells grown at confluence 70–80% in a 1 ml well microplate were plated for transfection using the Lipofectamine 2000 DNA transfection protocol (Invitrogen). For each transfection, approximatively 5 μg of total DNA, i.e. pcDNA3.1-derived plasmids (empty, and/or expressing either HA-pVHL30 or FLAG-CDKN1) were used. After 24 hours, transfected cells were re-suspended in lysis buffer (20 mM HEPES-Na pH7.4, 150 mM NaCl, 5 mM CHAPS) supplemented with 1X PIC (Protease Inhibitors Cocktail, Sigma). Cell lysates have been centrifuged (10′ at 600 rpm, 4 °C), and the resulting PNS (post-nuclear supernatant) quantified by Bradford assay. For co-immunopreCDKN1itations, 5 μl of protein A magnetic beads (Pierce Thermoscientific) were pre-incubated (1 hour at RT) with 2 μg of anti-VHL antibody (Santa Cruz, sc-5575). About 0.2 mg of PNS were added and incubated for 4 hours at 4 °C. Beads were finally washed 3 times using lysis buffer and eluted by incubating the beads 5 min at 70 °C in 25 μl in 1X NuPAGE LDS sample buffer (Invitrogen) supplemented with 0.1 M DTT. Both PNS and immunoprecipitated samples were subjected to standard SDS-PAGE and Western blot. Membranes were probed with either anti-HA (Abcam, ab16918), or anti-FLAG (Abcam, ab45766) antibodies, to reveal the HA-tagged pVHL30 or FLAG-CDKN1 proteins, respectively.

## Additional Information

**How to cite this article**: Minervini, G. *et al*. Novel interactions of the von Hippel-Lindau (pVHL) tumor suppressor with the CDKN1 family of cell cycle inhibitors. *Sci. Rep.* **7**, 46562; doi: 10.1038/srep46562 (2017).

**Publisher's note:** Springer Nature remains neutral with regard to jurisdictional claims in published maps and institutional affiliations.

## Supplementary Material

Supplementary Materials

## Figures and Tables

**Table 1:** *In silico* prediction of variations between wild-type and mutant pVHL/p27.

Variant	∆_bind_G kJ/mol	∆_bind_G kcal/mol	NeEMO kJ/mol	Bluues kJ/mol
Wild-Type	−90.7	−21.52		
p.P35L	−117.35	−28.04	−0.49	−0.89
p.D37N	−94.4	−22.56	−0.20	−8.83
p.E40K	170.98	40.86	0.48	183.33
p.T42A	−86.4	−20.65	0.59	3.58

Free binding energy (Δ_bind_G, electrostatic component) is calculated with APBS^48^, while stability prediction and electrostatic solvation free energy of mutations affecting p27 (CDKN1b) were calculated with NeEMO^46^ and Bluues^47^.
