# Supplementary material for: Novel interactions of the von Hippel-Lindau (pVHL) tumor suppressor with the CDKN1 family of cell cycle inhibitors
Source: Sci Rep. 2017 Apr 20;7:46562. doi: 10.1038/srep46562 (PMC5397843; doi:10.1038/srep46562)
Supplement: Supplementary Materials [file srep46562-s1.pdf]

Novel interactions of the von Hippel-Lindau (pVHL) tumor suppressor with the CDKN1 family of cell cycle inhibitors

Giovanni Minervini[1,*], Raffaele Lopreiato[1,*], Raissa Bortolotto[1], Antonella Falconieri[1], Geppo Sartori[1], Silvio C.E. Tosatto[1,2]

[1] Department of Biomedical Sciences, University of Padova, Viale G. Colombo 3, 35121, Padova, Italy.

[2] CNR Institute of Neuroscience, Padova, Viale G. Colombo 3, 35121, Padova, Italy.

[*] contributed equally

Supplementary Material

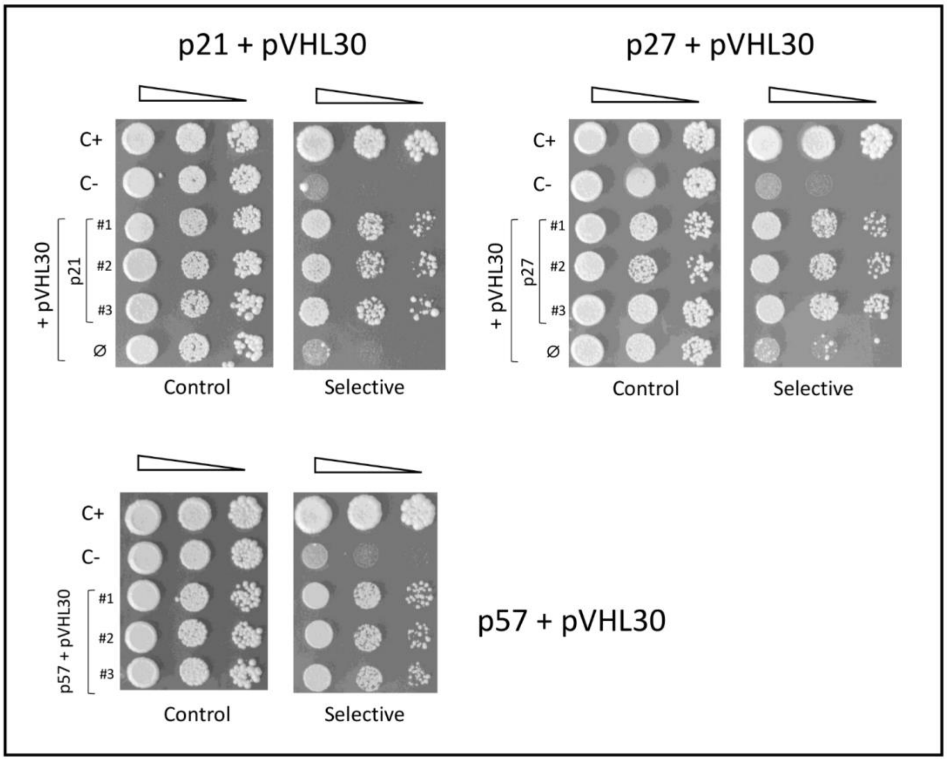

Supplementary Figure S1. pVHL is able to bind the CDKN1 proteins in a yeast two hybrid (Y2H) assay. The images are representative of 3 independent experiments, where 3 different clones co-expressing either p21, p27, or p57 with pVHL30 have been analysed by standard drop test. Cell growth on selective medium indicates positive interaction. Auto-activation by a Gal4BD-pVHL30 fusion protein has been excluded by the lethality of yeast cells co-transformed with the Gal4AD empty vector (∅). C+ and C- are positive and negative internal controls of the assay.

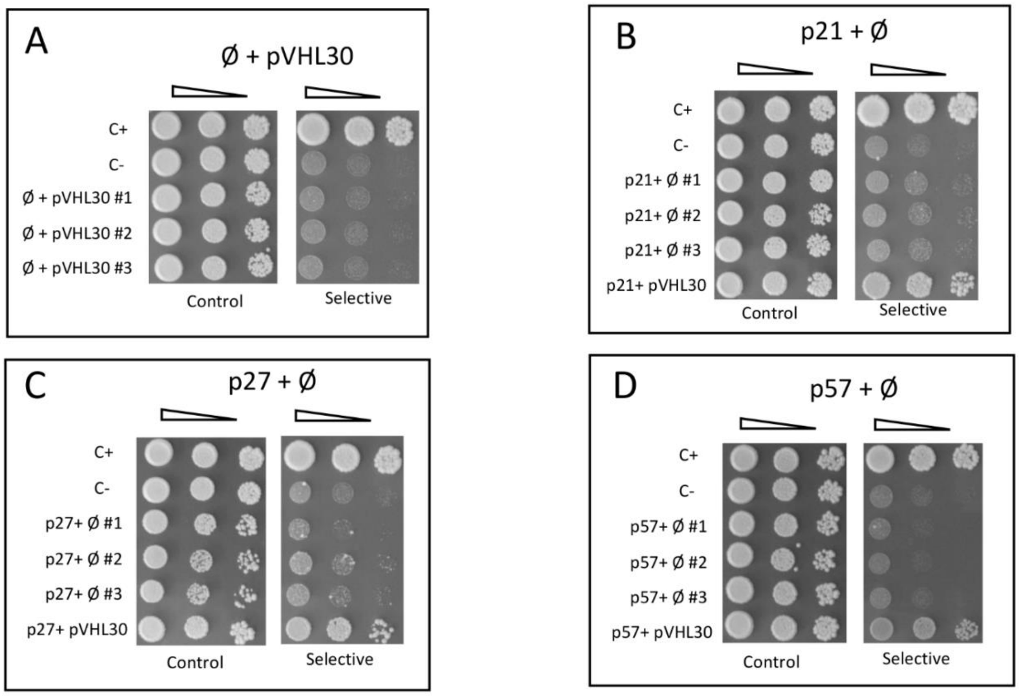

Supplementary Figure S2. Auto-activation by the pVHL30 and CDKN1 proteins in a Y2H assay.
The pVHL30 (**A**) and the CDKN1 (**B**: p21; **C**: p27; **D**: p57) proteins alone do not activate the reporter genes in a yeast two hybrid (Y2H) assay. Serial dilutions of yeast cells expressing either pVHL30 or CDKN1 (i.e. co-transformed with the empty vector, ∅) were spotted on both permissive (*left*) and selective (*right*) media, and incubated for several days at 30°C. Absence of cell growth on selective medium indicates lack of *HIS3* reporter gene activation. C+ and C- are positive and negative controls of the assay.

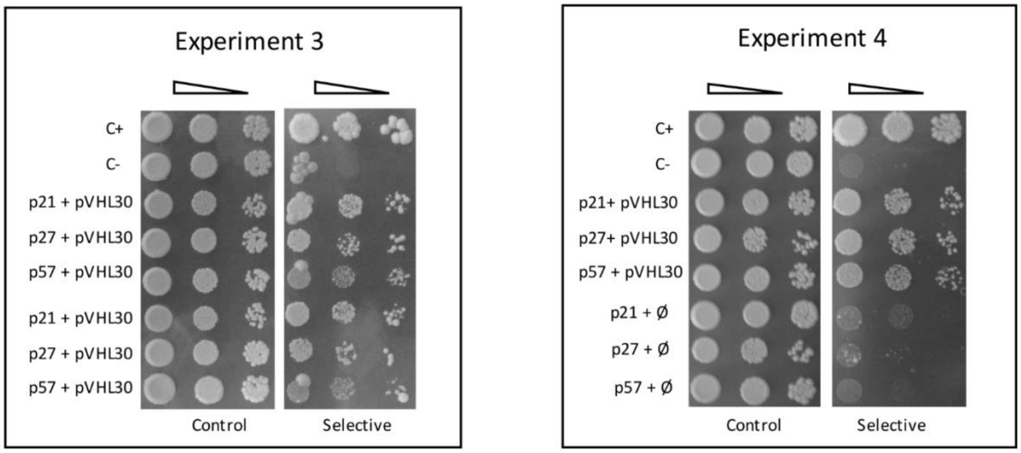

Supplementary Figure S3. pVHL is able to bind the CDKN1 proteins in a Y2H assay.
Results of two independent experiments (**A**: experiment 3; **B**: experiment 4) are shown, where different clones co-expressing either p21, p27, or p57 with pVHL30 have been assayed by drop test. Cell growth on selective medium indicates positive interactions. C+ and C- are positive and negative internal controls of the assay.

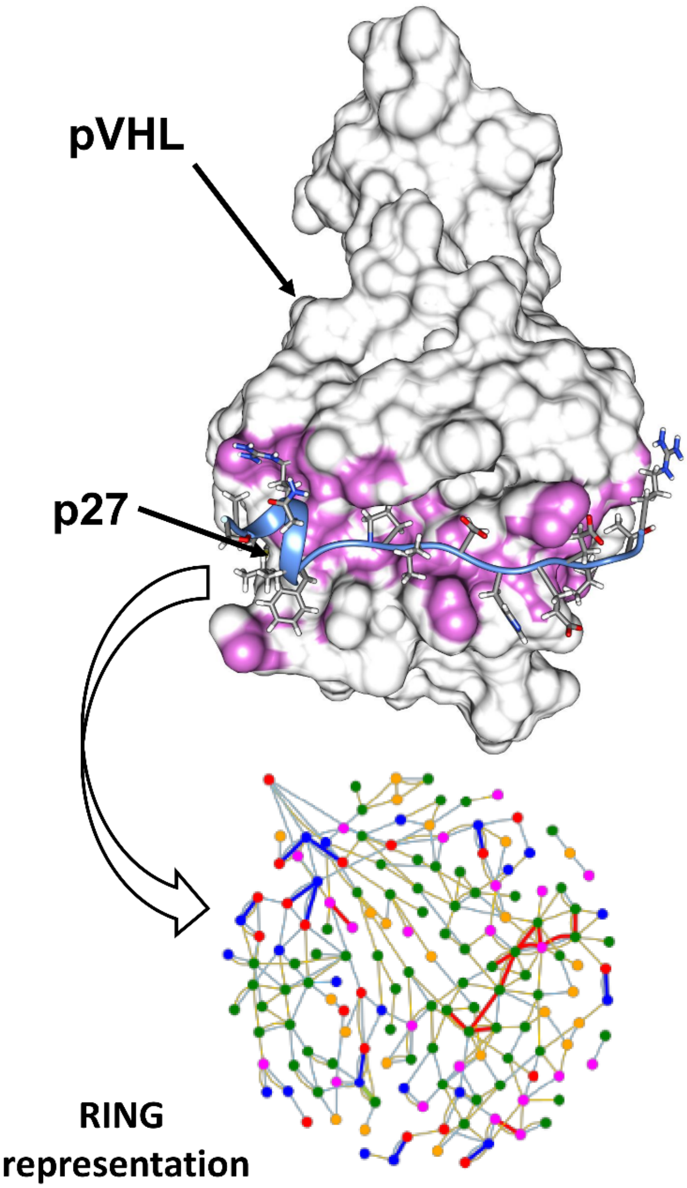

Residue p27	Type of interaction	Residue pVHL
H:29 CYS	VDW:MC_SC	V:91 PHE
H:30 ARG	VDW:SC_SC	V:90 ASN
H:30 ARG	HBOND:SC_SC	V:92 ASP
H:33 PHE	VDW:SC_SC	V:69 ARG
H:33 PHE	VDW:SC_SC	V:91 PHE
H:33 PHE	PIPISTACK:SC_SC	V:112 TYR
H:33 PHE	VDW:MC_SC	V:112 TYR
H:34 GLY	VDW:MC_SC	V:115 HIS
H:35 PRO	VDW:SC_SC	V:88 TRP
H:35 PRO	VDW:SC_SC	V:98 TYR
H:35 PRO	VDW:SC_SC	V:115 HIS
H:35 PRO	VDW:SC_SC	V:117 TRP
H:36 VAL	HBOND:MC_MC	V:110 HIS
H:36 VAL	VDW:SC_SC	V:110 HIS
H:37 ASP	VDW:SC_SC	V:109 ILE
H:38 HIS	HBOND:MC_SC	V:107 ARG
H:38 HIS	HBOND:MC_MC	V:108 ARG
H:38 HIS	VDW:SC_SC	V:108 ARG
H:38 HIS	PIPISTACK:SC_SC	V:110 HIS
H:38 HIS	VDW:SC_SC	V:110 HIS
H:39 ASP	IONIC:SC_SC	V:108 ARG
H:39 ASP	HBOND:SC_SC	V:108 ARG
H:39 ASP	VDW:SC_SC	V:108 ARG
H:40 GLU	HBOND:SC_SC	V:107 ARG
H:40 GLU	VDW:SC_SC	V:107 ARG
H:40 GLU	HBOND:MC_SC	V:108 ARG
H:41 LEU	VDW:SC_SC	V:75 ILE
H:41 LEU	VDW:SC_SC	V:77 CYS
H:41 LEU	HBOND:MC_MC	V:106 GLY
H:41 LEU	VDW:SC_MC	V:106 GLY
H:41 LEU	VDW:SC_SC	V:108 ARG
H:41 LEU	VDW:SC_SC	V:148 PHE
H:42 THR	VDW:SC_MC	V:106 GLY

Supplementary Figure S4. **Binding model of pVHL/p27 after 50 ns of MD simulations.**
Residues of pVHL forming the interaction surface are shown in purple and a cartoon representation is used for p27 (residues 21-42). Below the interaction network representing residues of interacting each other, while detailed description of interaction are presented on the right side.

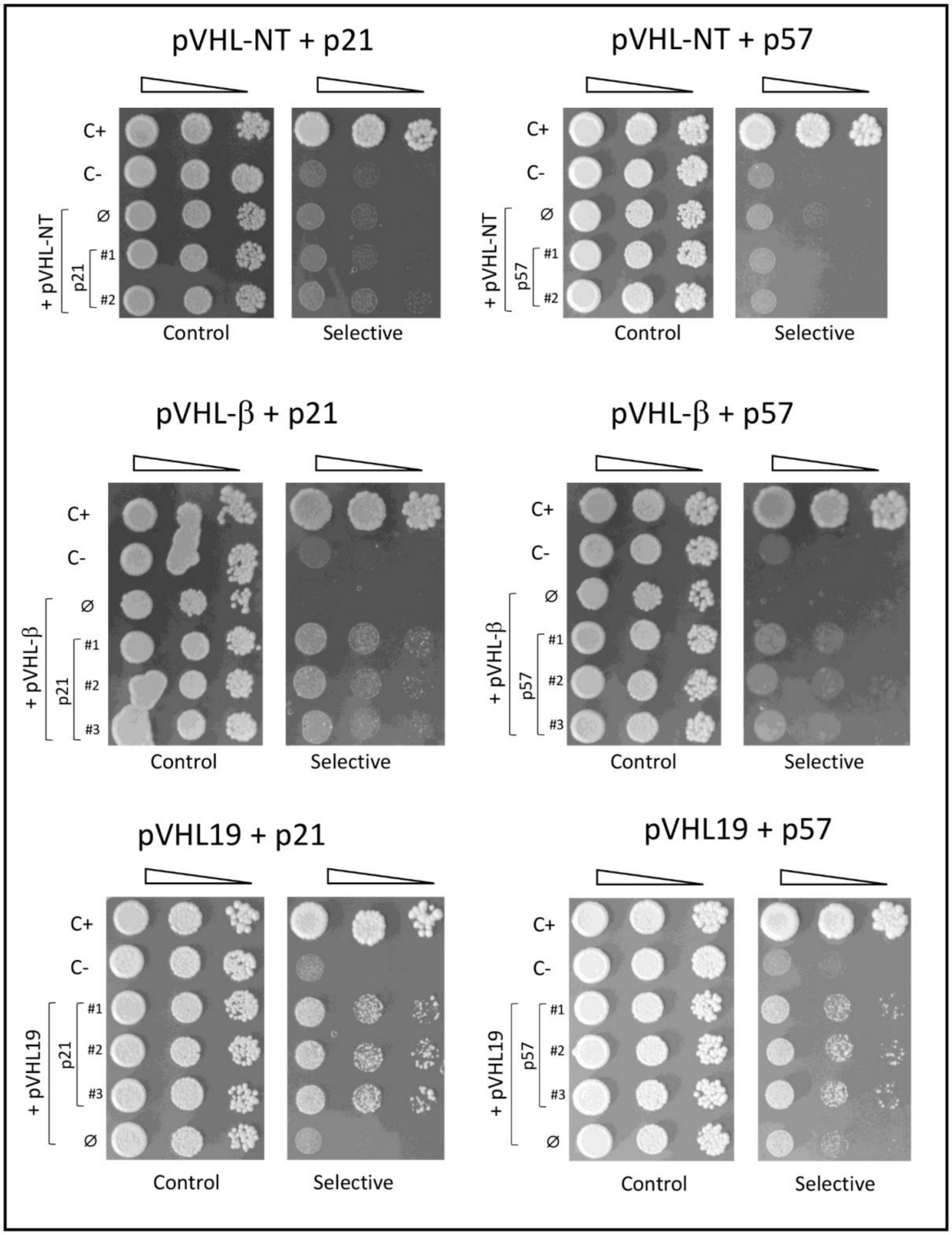

Supplementary Figure S5. pVHL dissection to map binding with p21 and p57 proteins.
(*Top*) The pVHL N-terminus has no role in binding p21 and p57. Yeast cells co-expressing pVHL-NT (residues 1-53) with either p21 and p57 (2 clones shown), or Gal4AD alone as control (empty vector, ∅), were tested for their binding in a Y2H assay. No cell growth on selective medium can be observed, indicating absence of interaction. (*Middle*) The pVHL-β region maintains binding with both p21 and p57. Yeast cells expressing pVHL-β (aa 54-157) together with either p21 and p57 are able to grow in selective medium, whereas cells expressing pVHL-β alone (empty vector, ∅), cannot survive, supporting that the β-domain is sufficient for binding. Three independent clones for each CDKN1 are shown. (*Bottom*) The pVHL19 isoform interacts with p21 and p57. Yeast cells co-expressing pVHL19 (residues 54-213) with either p21 and p57 (3 independent clones each CDKN1) were spotted on permissive and selective medium, where yeast growth is indicative of their binding. Absence of growth for pVHL19 alone (empty vector, ∅) is also shown, excluding aspecific reporter activation.

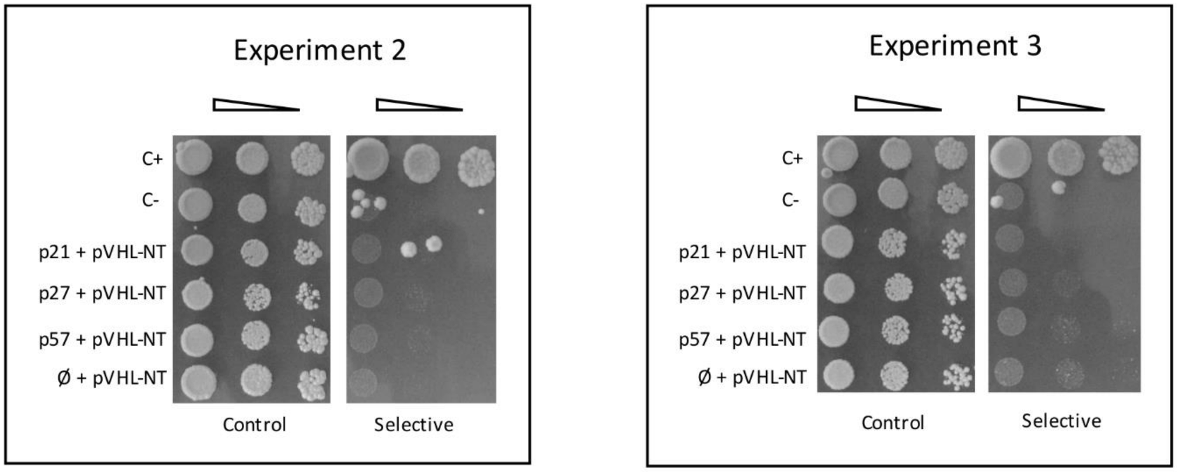

Supplementary Figure S6. CDKN1/pVHL-NT Y2H assays.
Results of two independent experiments (**A**: experiment 2; **B**: experiment 3) are shown, where different clones co-expressing either p21, p27, or p57 with pVHL-NT have been assayed by drop test. Absence of yeast cell growth on selective medium indicates lack of interaction. C+ and C- are positive and negative internal controls of the assay.

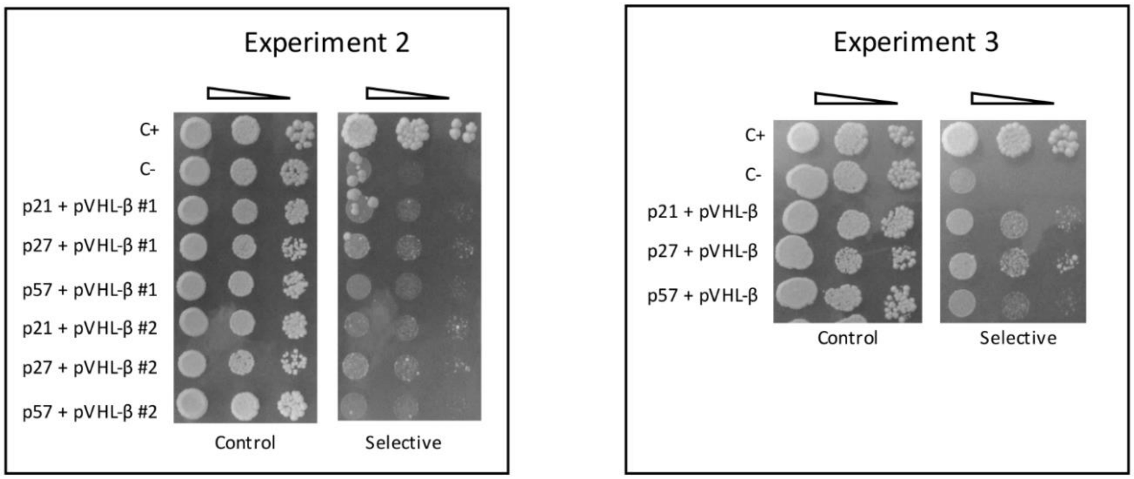

Supplementary Figure S7. CDKN1/pVHL-β Y2H assays.
Results of two independent experiments (**A**: experiment 2; **B**: experiment 3) are shown, where different clones co-expressing either p21, p27, or p57 with pVHL-β have been assayed by drop test. Growth of yeast cells on selective medium indicates the ability of the proteins to interact. C+ and C- are positive and negative internal controls of the assay.

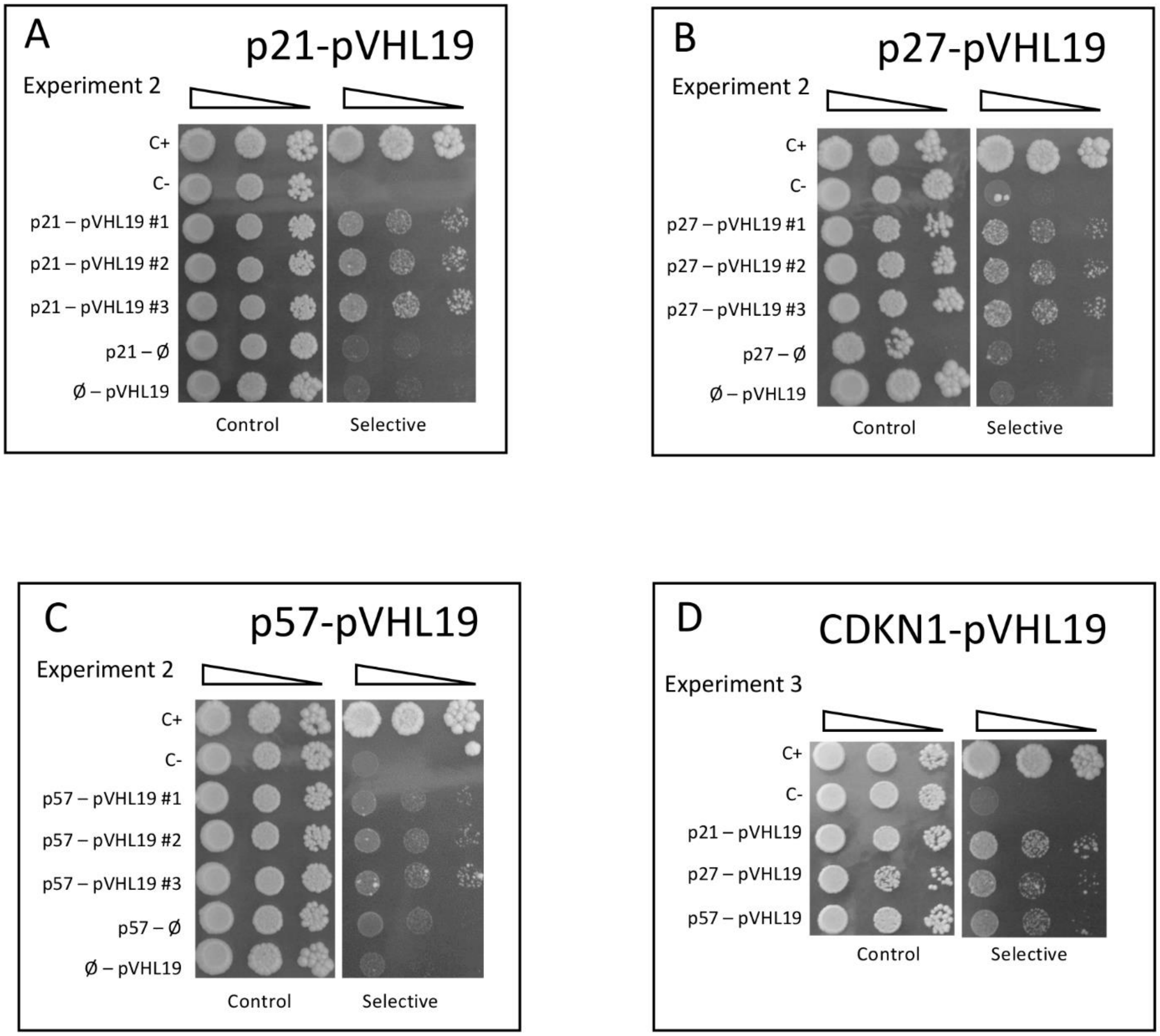

Supplementary Figure S8. CDKN1/pVHL19 Y2H assays.
Results of two independent experiments (**A-C**: experiment 2; **D**: experiment 3) are shown, where different clones co-expressing either p21 (**A,D**), p27 (**B,D**), or p57 (**C,D**) with pVHL19 have been assayed by drop test. Growth of yeast cells on selective medium indicates the ability of the proteins to interact. C+ and C- are positive and negative internal controls of the assay.

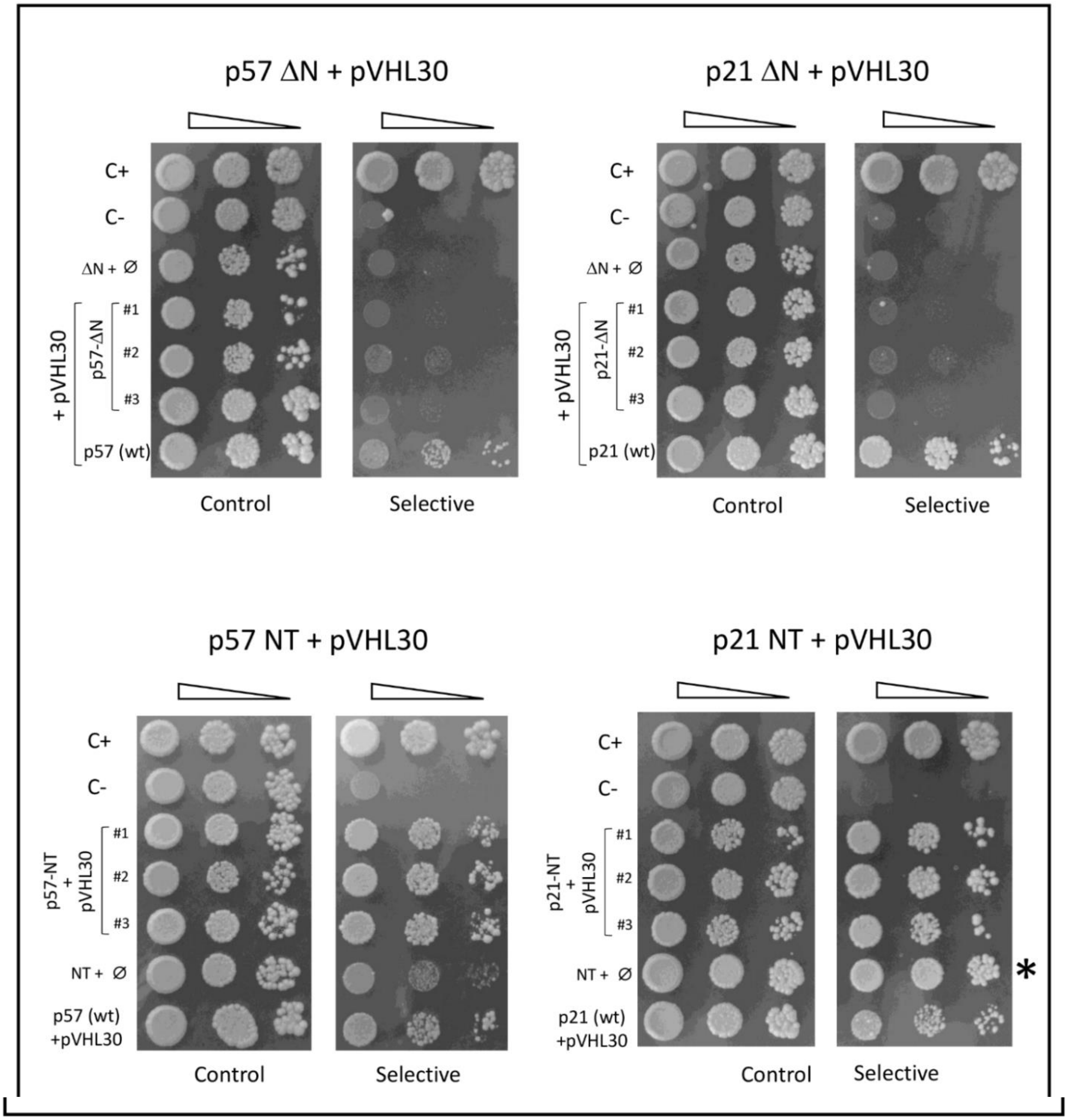

Supplementary Figure S9. p57 and p21 dissection to map binding with pVHL.
(*Top*) The N-terminal tail of the p57 and p21 proteins is essential for binding. Removal of the p57 and p21 N-terminus (ΔN, residues 1-60 and 1-49, respectively) abolishes interaction with pVHL30. No growth in selective medium is observed for 3 clones of yeast cells co-expressing pVHL30 and ΔN mutant proteins, unlike the full-length sequences (wt). Plates were incubated at 30°C for more time (8 days) to confirm the absence of yeast growth. (*Bottom*) The N-terminal region the p57 protein is able to bind pVHL. Yeast cells expressing pVHL30 together with the p57 N-terminus (NT, residues 1-60) were tested for binding by Y2H assay. On selective medium, yeast cells expressing p57-NT grow better than cells carrying the full-length protein (wt). Plates were incubated at 30 °C for 5 days. Data on p21-NT (aa 1-49) cannot be considered, due to its ability to activate the *HIS3* reporter gene in presence of the empty vector (∅), as indicated by the asterisk (*). In all panels, C+ and C- are positive and negative controls of the assay. The images are representative of at least 3 independent experiments, where 2-4 different clones were analyzed.

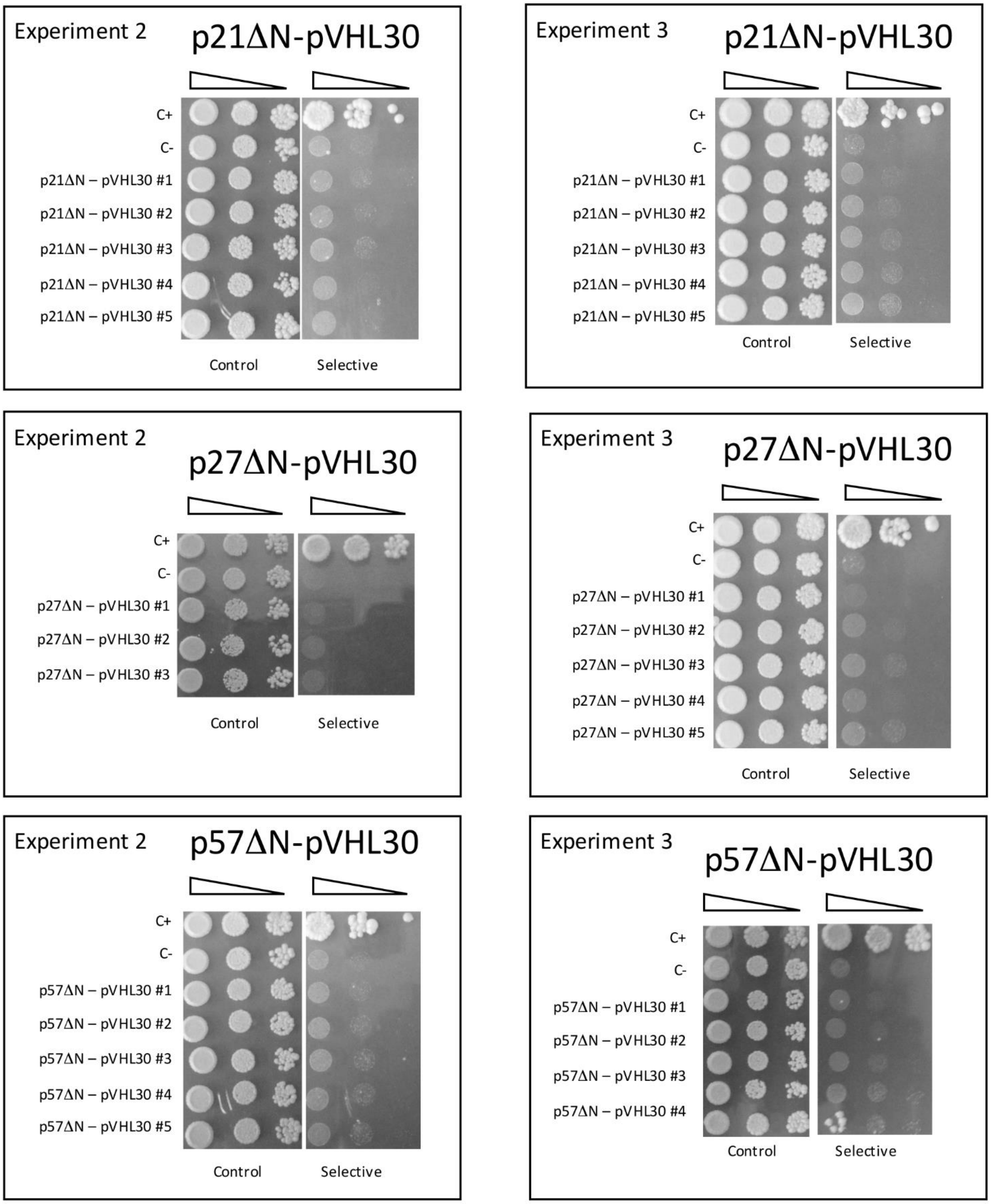

Supplementary Figure S10. CDKN1-ΔN/pVHL30 Y2H assays.
Results of two independent experiments (**A**: experiment 2; **B**: experiment 3) are shown, where different clones co-expressing either p21-ΔN (*top*), p27-ΔN (*middle*), or p57-ΔN (*bottom*) with pVHL30 have been assayed by drop test. Growth of yeast cells on selective medium indicates the ability of the proteins to interact. C+ and C- are positive and negative internal controls of the assay.

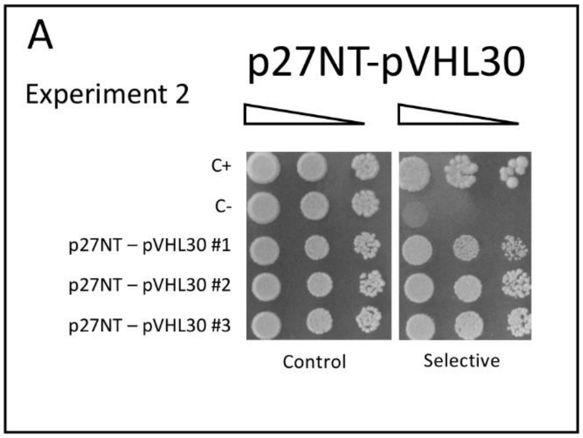

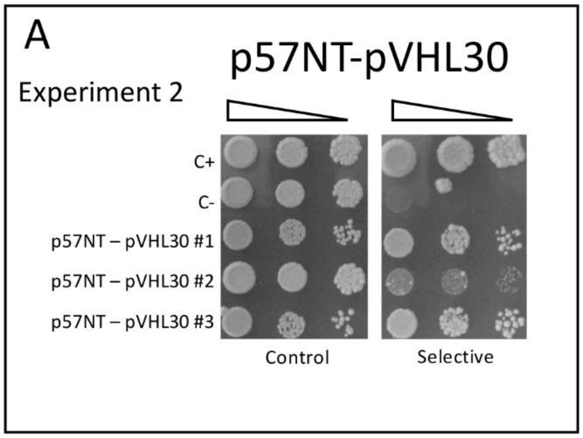

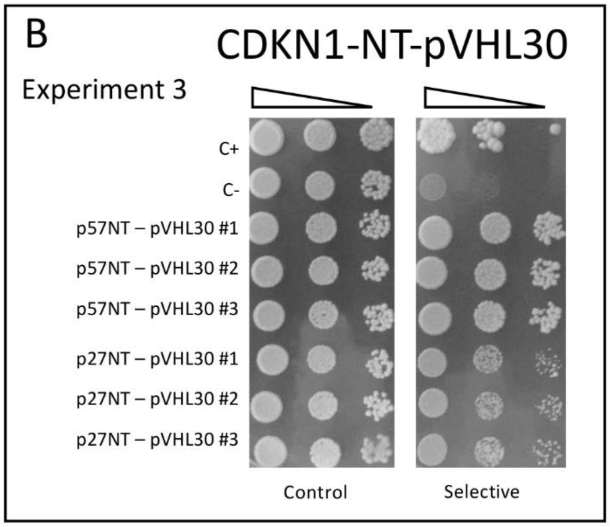

Supplementary Figure S11. CDKN1-NT/pVHL30 Y2H assays.
Results of two independent experiments (**A**: experiment 2; **B**: experiment 3) are shown, where different clones co-expressing either p27-NT or p57-NT with pVHL30 have been assayed by drop test. Growth of yeast cells on selective medium indicates the ability of the proteins to interact. C+ and C- are positive and negative internal controls of the assay.

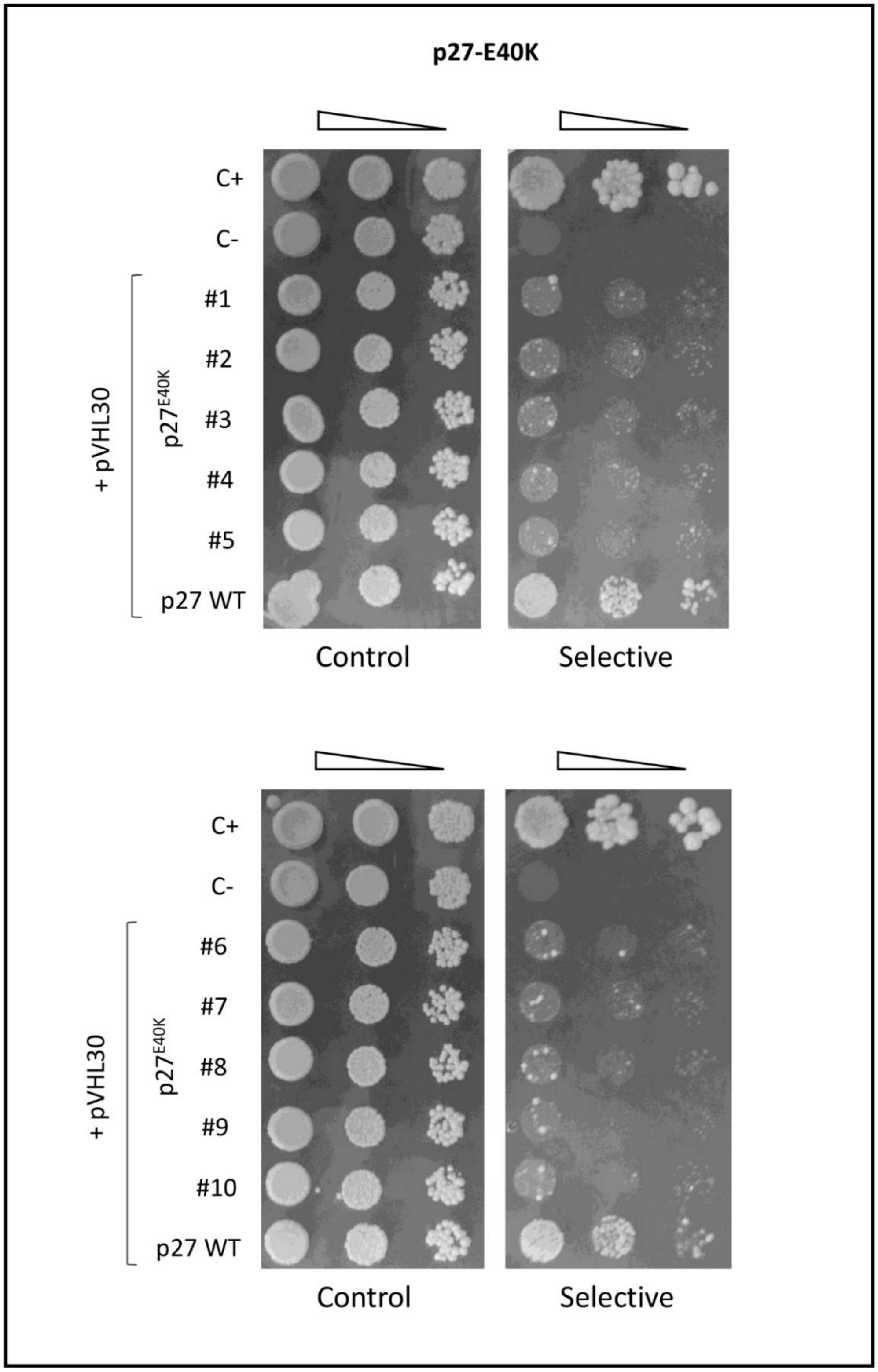

Supplementary Figure S12. Negative effect of the p27 E40K mutation on pVHL binding.
Multiple clones of yeast cells co-expressing pVHL30 and the p27 E40K mutant isoform were assayed for binding. Cells have been grown in selective medium supplemented with 60 mM 3AT, in order to increase the stringency of the binding assay. In all experiments, C+ and C- are positive and negative controls of the assay. The images are from two independent experiments (top and bottom), where 5 clones were analyzed.

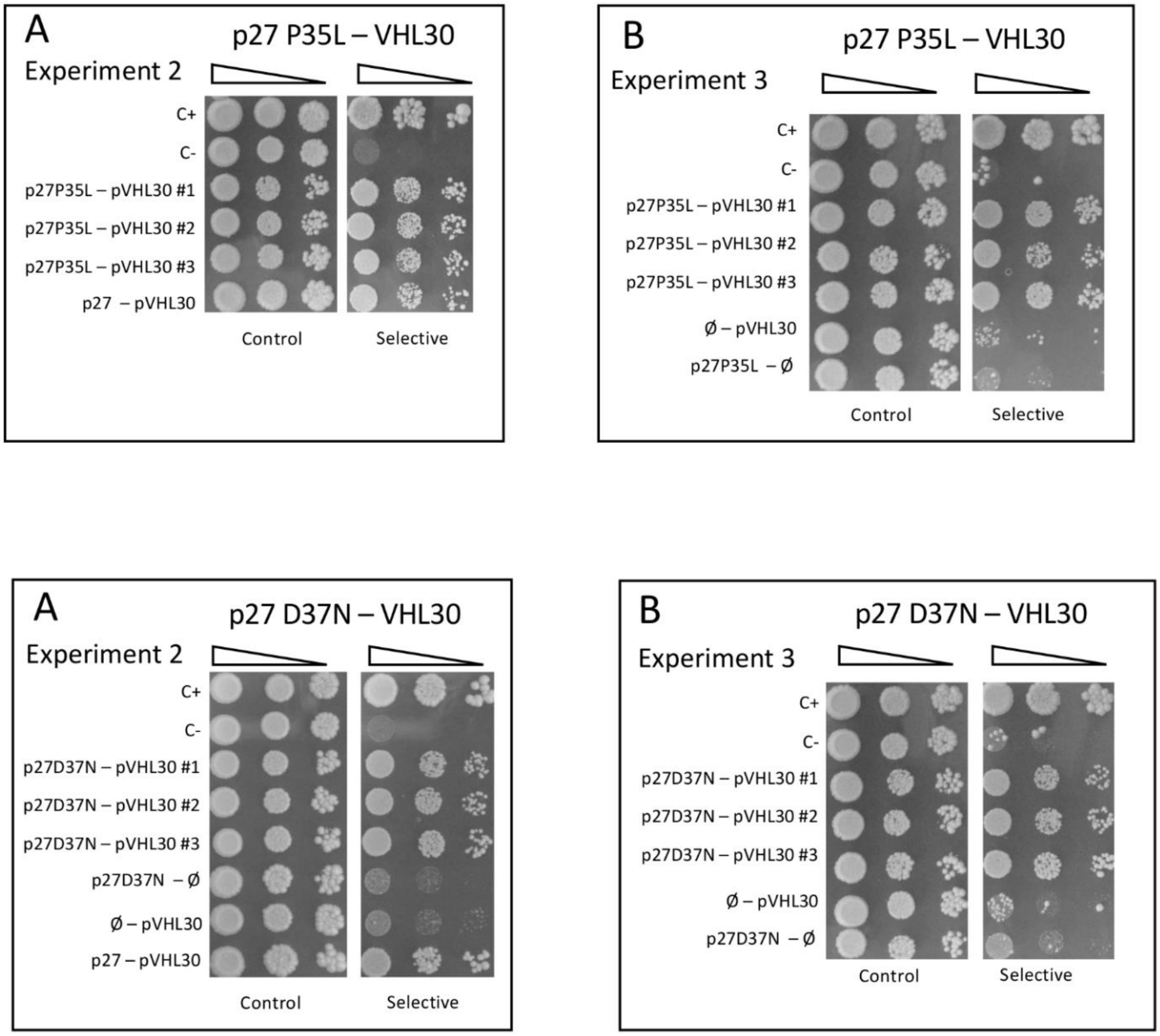

Supplementary Figure S13. p27 mutant/pVHL30 Y2H assays.
Yeast cells co-expressing pVHL30 together with the indicated mutant p27 isoforms (P35L, *top*; D37N, *bottom*) were assayed for binding. Results of two independent experiments are shown (**A**: experiment 2; **B**: experiment 3). C+ and C- are positive and negative controls of the assay.

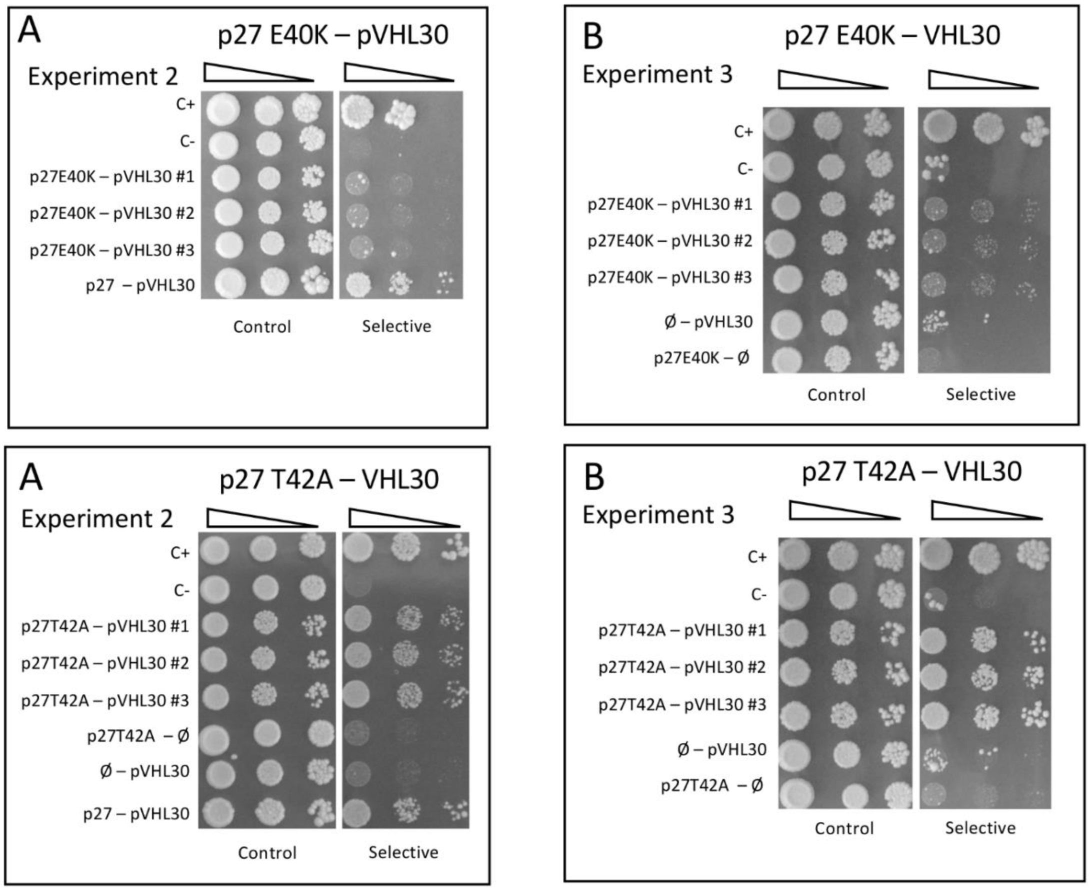

Supplementary Figure S14. p27 mutant/pVHL30 Y2H assays.
Yeast cells co-expressing pVHL30 together with the indicated mutant p27 isoforms (E40K, *top*; T42A, *bottom*) were assayed for binding. Results of two independent experiments are shown (**A**: experiment 2; **B**: experiment 3). C+ and C- are positive and negative controls of the assay.

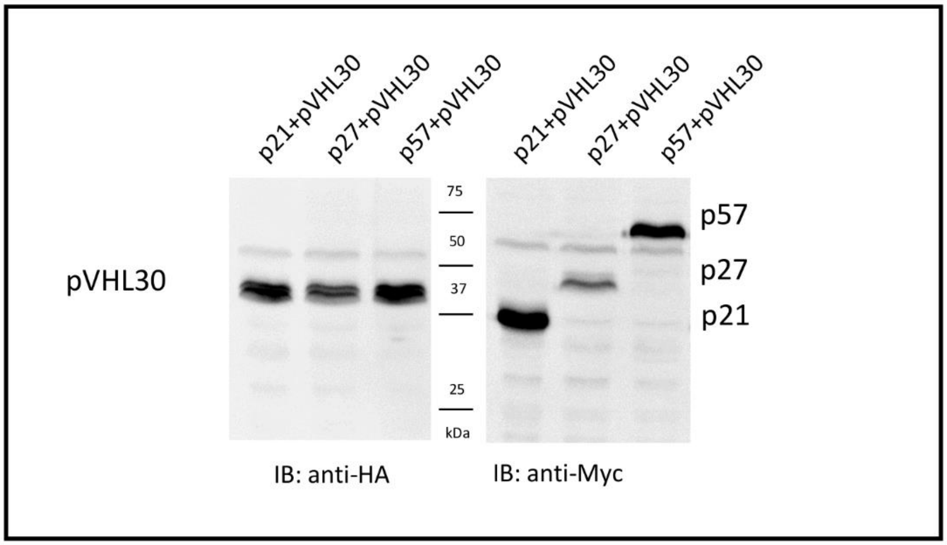

Supplementary Figure S15. Western blot analysis of total protein lysates.
Yeast cells co-expressing pVHL30 (HA-tagged), and each CDKN1 (Myc-tagged), as Gal4-fusion proteins are shown. Membranes were immunoblotted with either anti-HA (*left*), and anti-Myc (*right*) antibodies, revealing the presence of fusion proteins at the expected molecular weight, as indicated. *Lane 1*: p21+pVHL30; *lane 2*: p27+pVHL30; *lane 3*: p57+pVHL30.

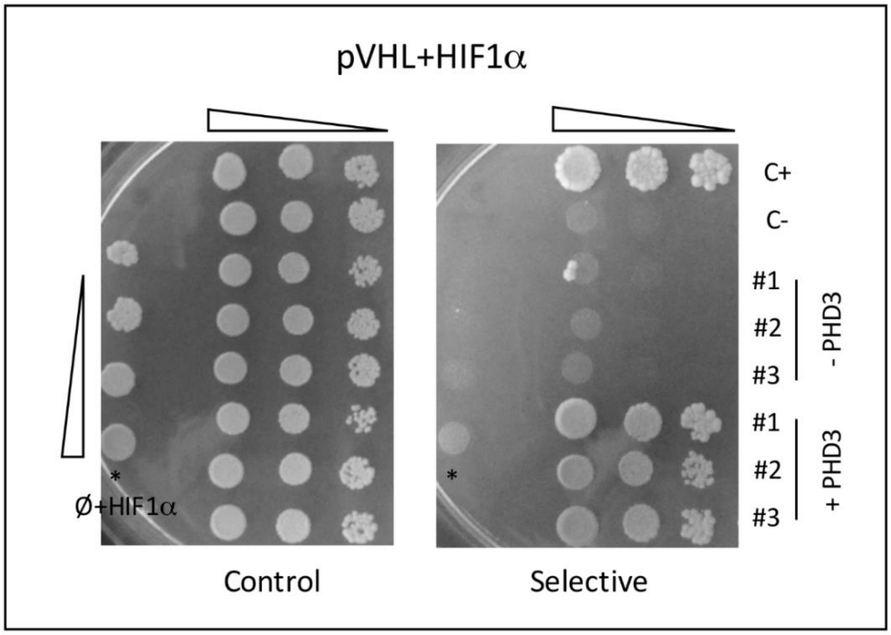

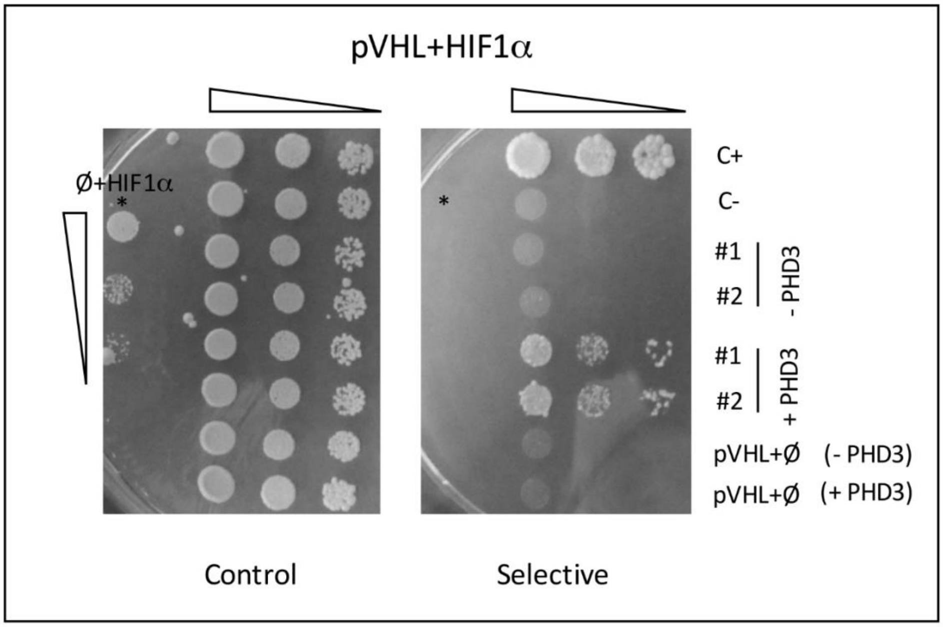

Supplementary Figure S16. pVHL30/HIF-1a binding by Y2H assay.

(*Top*) Proline hydroxylation of HIF-1α (P402 and P564) by the PHD3 enzyme is required for pVHL binding. Lack of PHD3 abolishes the interaction, as no growth in selective medium is observed for the yeast cells co-expressing only pVHL and HIF-1α proteins. Auto-activation of Gal4AD-HIF-1α fusion proteins can be excluded by the lethality of the yeast cells co-transformed with an empty Gal4BD vector (marked *). (*Bottom*) A second independent experiment has been performed, where autoactivation by both pVHL30 alone or together with PHD3 has been excluded by the lethality of the yeast cells co-transformed with an empty Gal4AD vector. In both panels, C+ and C- are positive and negative controls of the assay. Y2H bait plasmids have been obtained by cloning the coding sequence of pVHL30 into the EcoRI/BamHI sites of MCSI, and the full-length PHD3 into NotI/BglII sites of MCS II of pBridge vector (Clontech), as described (Bex C. et al., Nucleic Acids Research 2007, 35:e142). The Y2H prey plasmid has been constructed by cloning the coding sequence of full-length HIF-1α into the pGADT7 vector (Clontech).

Supplementary Table S1. Primer list. Primer sequences are in 5' to 3' direction. The 15 nt-long recombinogenic regions required for cloning with the In-fusion method corresponding to the plasmid sequences surrounding the EcoRI site present both in the pGBKT7 and pGADT7 plasmids are indicated in lowercase letters. The EcoRI site is underlined and was removed in the reverse primer by substitution of the first G with a C. (*) indicates that the same primer was used in multiple cloning reactions.

Plasmid Name	Primer Name	sequence (5'-3')	Protein expressed
pVHL30-pGAD	VHL30-AD For (*)	ggaggccagtgaattcATGCCCCGGAGGGCGGAGAA	Gal4AD-pVHL30 (1-213)
	VHL30-AD Rev	cacccgggtggaattgTCAATCTCCCATCCGTTGAT	
pVHL-NT-pGAD	VHL30-AD For (*)		Gal4AD-pVHL-NT (1-53)
	VHLNT-AD Rev	cacccgggtggaattgTCACTCCTCCTCGGCGCCCA	
pVHL-Δ-pGAD	VHL19-AD For	ggaggccagtgaattcATGGAGGCCGGGCGGCCGCG	Gal4AD-pVHL-Δ (54-157)
	VHLb-AD Rev	cacccgggtggaattgCTAAGTATACACTGGCAGTGTGATATTGGC	
pVHL19-pGAD	VHL19-AD For (*)		Gal4AD-pVHL19 (54-213)
	VHL30-AD Rev (*)		
p21-pGBK	p21-BD For	catggaggccgaattcATGTCAGAACCGGCTGGGGATGTCC	Gal4BD-p21 (1-164)
	p21-BD Rev	ggatccccgggaattgCTA GGGCTTCCTCTTGGAGAAGATCAGC	
p21-NT-pGBK	p21-BD For (*)		Gal4BD-p21-NT (1-49)
	p21NT-BD Rev	ggatccccgggaattgCTACCATCGCTCACGGGCCTCCTGGATGC	
p21-ΔN-pGBK	p21CT-BD For	catggaggccgaattcAACTTCGACTTTGTCACCGAGACACC	Gal4BD-p21-DN (50-164)
	p21-BD Rev (*)		
p27-pGBK	p27-BD For	catggaggccgaattcATGTCAAACGTGCGAGTGTC	Gal4BD-p27 (1-198)
	p27-BD Rev	ggatccccgggaattgTCACGTTTGACGTCTTCTGAG	
p27-NT-pGBK	p27-BD For (*)		Gal4BD-p27-NT (1-60)
	p27NT-BD Rev	ggatccccgggaattgCTACCACTTGCGCTGGCTCGCCTCTTCC	
p27-□N-pGBK	p27CT-BD For	catggaggccgaattcAATCACAAACCCCTAGAGGGCAAGTACG	Gal4BD-p27-ΔN (61-198)
	p27CT-BD Rev	ggatccccgggaattgCATGGTGGCGGATCCGAGCTCGGTAC	

		C	
p57-pGBK	p57-BD For	catggaggccgaattcATGTCCGACGCGTCCCTCCG	Gal4BD-p57 (1-316)
	p57-BD Rev	ggatccccgggaattgTCACCGCAGCCTCTTGCGCG	
p57-NT-pGBK	p57NT-BD For	catggaggccgaattcATGTCCGACGCGTCCCTCCGCAGC	Gal4BD-p57-NT (1-61)
	p57NT-BD Rev	ggatccccgggaattgCTACCAGCGGTTCTGGTCCTCGGCGTTC	
p57-□N-pGBK	p57CT-BD For	catggaggccgaattcGATTACGACTTCCAGCAGGACATGC	Gal4BD-p57-ΔN (62-316)
	p57CT-BD Rev	ggatccccgggaattgCTACCGCAGCCTCTTGCGCGGGGTCTGC	
P35L-p27-pGBK	P35L-p27 For	CTTCGTGGTCCACCAGGCCGAAGAGGTTC	Gal4BD-p27-P35L
	P35L-p27 Rev	GAACCTCTTCGGCCTGGTGGACCACGAAG	
D37N-p27-pGBK	D37N-p27 For	TTAACTCTTCGTGGTTCACCGGGCCGAAGAG	Gal4BD-p27-D37N
	D37N-p27 Rev	CTCTTCGGCCCGGTGAACCACGAAGAGTTAA	
E40K-p27-pGBK	E40K-p27 For	CCGGGTTAACTTTTCGTGGTCCACCGGG	Gal4BD-p27-E40K
	E40K-p27 Rev	CCCGGTGGACCACGAAAAGTTAACCCGG	
T42A-p27-pGBK	T42A-p27 For	AAGTCCCGGGCTAACTCTTCGTGGTCCACC	Gal4BD-p27-T42A
	T42A-p27 Rev	GGTGGACCACGAAGAGTTAGCCCGGGACTT	
pVHL30-pcDNA3.1	HA-VHL For	taccgagctcggatcATGGAGTACCCATACGACGTACCAGATTACG	HA-pVHL30 (1-213)
	HA-VHL Rev	gatatctgcagaattTCAATCTCCCATCCGTTGATGTGCAATGCGC	
p21-pcDNA3.1		GeneScript cat n: OHu26670	Flag-p21 (1-164)
p27-pcDNA3.1		GeneScript cat n: OHu27895	Flag-p27 (1-198)
p57-pcDNA3.1		GeneScript cat n: OHu27234	Flag-p57 (1-316)
